# Supplementary material for: Mutational interactions define novel cancer subgroups
Source: Nat Commun. 2018 Oct 19;9:4353. doi: 10.1038/s41467-018-06867-x (PMC6195543; doi:10.1038/s41467-018-06867-x)
Supplement: Supplementary file 1 — Supplementary Information [file 41467_2018_6867_MOESM1_ESM.pdf]

# Supplementary material:
# Mutational interactions define novel cancer subgroups

Jack Kuipers[1,2]*, Thomas Thurnherr[1], Giusi Moffa[3,4], Polina Suter[1,2], Jonas Behr[1], Ryan Goosen[5], Gerhard Christofori[5] and Niko Beerenwinkel[1,2]*

[1]*Department of Biosystems Science and Engineering, ETH Zurich, Basel, Switzerland*
[2]*SIB Swiss Institute of Bioinformatics, Basel, Switzerland*
[3]*Division of Psychiatry, University College London, London, UK*
[4]*Institute for Clinical Epidemiology and Biostatistics, University Hospital Basel, Basel, Switzerland*
[5]*Department of Biomedicine, University of Basel, Basel, Switzerland*

# Supplementary methods

## A Bayesian network modelling

Our Bayesian network modelling relates aspects of patient sample clustering, tumour progression, and mutational interactions. Here we provide a brief overview of these topics.

**A.1 Pan-cancer clustering** The TCGA has collected and made publicly available data from tissue samples from more than 30 different cancers. A subset of 12 cancer types with more than 3000 samples have previously been extensively investigated. Specifically, samples were organised by hierarchical clustering of gene level copy number changes, mutations and methylation events [1], thereby highlighting the differences in terms of functional events, altered processes, and pathways. For the same cancer types (but slightly different patient samples), the patient samples were clustered, by integrating different data types: DNA methylation and sequencing, copy-number variations, and mRNA, microRNA and protein levels [2]. Strong signatures of the tissue of origin were found, but roughly 10% of the samples were regrouped. The implied presence of molecular subtypes spanning diverse tissue types could be highly therapeutically relevant. Reclustering may potentially enable cancer patients to access a wider array of existing anti-cancer therapeutics and enable repurposing and repositioning of existing drugs across different subtypes beyond their cancer-type indications.

**A.2 Cancer progression modelling** Along with clustering patient samples based on their mutational profile, we can also view each sample as a snapshot of an evolutionary process where the accumulation of heritable alterations enhance the capabilities of the cancer tissue that allow tumour progression [3, 4]. Early models for a cancer progression mechanism considered a linear sequential build-up of advantageous mutations [5]. The simple linear progression was extended to tree models [6], called oncogenetic trees, to allow for branching and concurrent tumour evolution. Sample heterogeneity was accounted for by mixtures of trees [7]. A more complex and general class of cancer progression models is provided by Conjunctive Bayesian Networks (CBNs) [8] which allow for converging lineages. CBNs are waiting time models

*to whom correspondence should be addressed: jack.kuipers@bsse.ethz.ch; niko.beerenwinkel@bsse.ethz.ch

where each mutation must wait for its parents in the network to occur before having a chance to proceed, with any violations assumed to be errors in the data [9]. Pathway progression models have also been compared [10]. An alternative approach to CBNs allows any transition between different mutations and then infers the most likely sequence of events [11].

Both oncogenetic trees and CBNs act on a subspace of directed acyclic graphs (DAGs) enforcing strict temporal ordering along the edges. A consequence of tumour heterogeneity though is that different patient samples may not necessarily experience the same order. Which genomic alterations are beneficial for tumour progression at any given time-point depends on the micro environment of the cancer cells as well as on the genetic background of previous alterations. Rather than considering strict ordering, further progression models have also been developed for large scale cross-sectional data like the TCGA database, involving both tree [12] and DAG based Bayesian networks [13, 14]. We focus on uncovering the dependence structure among mutations by relaxing our model space to the entire set of DAGs and treating the mutations as a Bayesian network, accounting for mutational interactions.

**A.3 Co-occurrence and mutual exclusivity** Co-occurrence between mutations will lead to strong edges in the network, as will mutual exclusivity, which may point to mutations acting on the same pathway. The idea is that only the first disruption in a pathway leads to a fitness advantage for the tumour cells, and to clonal expansion. Further mutations may have only little additional effect and not be selected for. Therefore each patient sample would be expected to only display the first mutation. Methods have been developed [15, 16, 17] to detect the resulting mutual exclusive patterns across patients samples as well as mutually exclusive subnetworks in pan-cancer data [18] or to integrate data to uncover synthetic lethality [19], a more extreme version of exclusivity where additional mutations along the same pathway actually lead to a fitness disadvantage. Mimicking the effect of these mutations may constitute a therapeutic strategy. Mutual exclusivity discovery has also been combined with CBN progression models to infer the temporal ordering of cancer pathways [20].

Co-occurence on the other hand may result from a fitness valley, where single mutations are selected against but combinations have a fitness advantage. This might indicate an interactive mechanism between the co-mutated genes relevant for cancer progression. Mutations may not directly cause each other but may influence the probability that a new mutation fixates through mechanistic changes which promote or inhibit the selection of further mutations. Functional interactions are abundant so that dependencies at the level of mutations are to be expected. A causal interpretation of the networks we learn from mutation profiles would therefore need to be viewed through the lens of fitness. In this way, each aberration in the network might influence downstream mutation states.

Supplementary Table 1: TCGA pan-cancer data set. Abbreviations: basal – Basal-like; her2 – Her2 enriched; lumA – Luminal A; lumB – Luminal B; MSI – Microsatellite instability; MSI-H – MSI-high; MSI-L – MSI-low; MSS – Microsatellite stable.

| Tumor type (Subtypes) | TCGA ID | Number of cases | Reference |
|---|---|---|---|
| Bladder urothelial carcinoma | blca | 395 | [21] |
| Breast invasive carcinoma (basal, her2, lumA, lumB) | brca | 977 | [22, 23] |
| Cervical squamous cell carcinoma and endocervical adenocarcinoma | cesc | 194 | [24] |
| Colon and rectal adenocarcinoma (MSI, MSI-H, MSI-L, MSS) | coadread | 489 | [25] |
| Esophageal carcinoma | esca | 185 | [24] |
| Glioblastoma multiformae (classical, mesenchymal, neural, proneural) | gbm | 283 | [26] |
| Head and neck squamous cell carcinoma | hnsc | 511 | [27] |
| Kidney renal clear-cell carcinoma | kirc | 436 | [28] |
| Kidney renal papillary cell carcinoma | kirp | 282 | [24] |
| Acute myeloid leukemia | laml | 193 | [29] |
| Brain lower grade glioma | lgg | 516 | [30] |
| Liver hepatocellular carcinoma | lihc | 373 | [24] |
| Lung adenocarcinoma (bronchioid, magnoid, squamoid) | luad | 533 | [31] |
| Lung squamous cell carcinoma | lusc | 178 | [32] |
| Ovarian serous cystadenocarcinoma | ov | 466 | [33] |
| Pancreatic adenocarcinoma | paad | 126 | [24] |
| Pheochromocytoma and paraganglioma | pcpg | 179 | [24] |
| Prostate adenocarcinoma | prad | 498 | [24] |
| Sarcoma | sarc | 247 | [24] |
| Stomach adenocarcinoma | stad | 393 | [24] |
| Thyroid carcinoma (classical, follicular, tall-cell) | thca | 496 | [24] |
| Uterine corpus endometrioid carcinoma (MSI-H, MSI-L, MSS) | ucec | 248 | [34] |

## B Improvement in network inference

Learning graphical models is highly challenging for large networks. Here we perform a simulation study to show the advantages of our method over alternatives. We compared to the PC algorithm [35] as implemented in the **pcalg** package [36] and greedy equivalence search [37]. The performance of greedy equivalence search in the setting was notably poorer, so we focus on the comparison with the PC algorithm. First, 50 random power-law DAGs were generated with 100 nodes. For compatibility with the TCGA data, the DAGs had an expected number of edges of 100 and marginal node frequencies which followed the same distribution as the TCGA mutational data. For each DAG, 200 and 400 binary mutation profiles were generated in line with the numbers of sequenced tumours per cancer type in TCGA data. DAGs were then inferred with the PC algorithm from **pcalg** and sampled with our MCMC method **BiDAG** [38] with an edge penalisation of 16 and compared to the generating DAGs. The number of true and false positive edges in the network skeleton were tracked as the significance threshold was varied for the PC algorithm or as the posterior threshold of edge probabilities was varied for our MCMC scheme (Supplementary Figure 1). Along with uncovering the best performance with a posterior threshold of $0.5$, our method provides notably more accurate network inference. Even with a small sample size, we find the vast majority of edges in the true network unlike the PC algorithm which discovers less than half. With the larger sample size of 400, our inferred networks are very close to the true ones. Our method also characterises the full posterior distribution of networks, unlike the point estimates provided by the PC algorithm or greedy equivalence search.

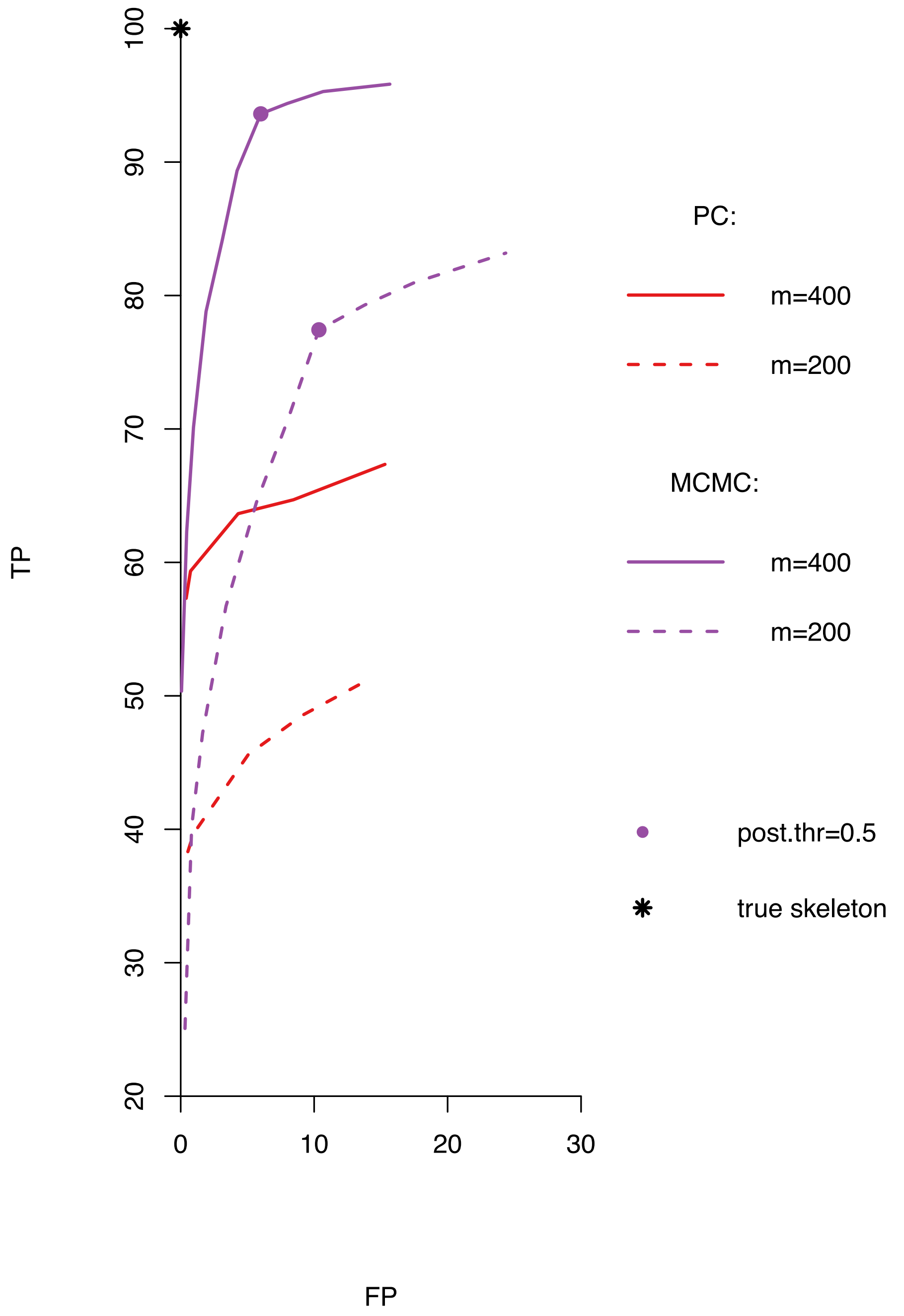


Supplementary Figure 1: The accuracy of inferring the network structure in terms of the true positive (TP) and false positive (FP) edges in the underlying skeleton. The number of observations, $m$, was set to 200 and 400 and they were generated for networks of 100 nodes with 100 edges on average. The PC algorithm performs notably worse than our MCMC scheme.

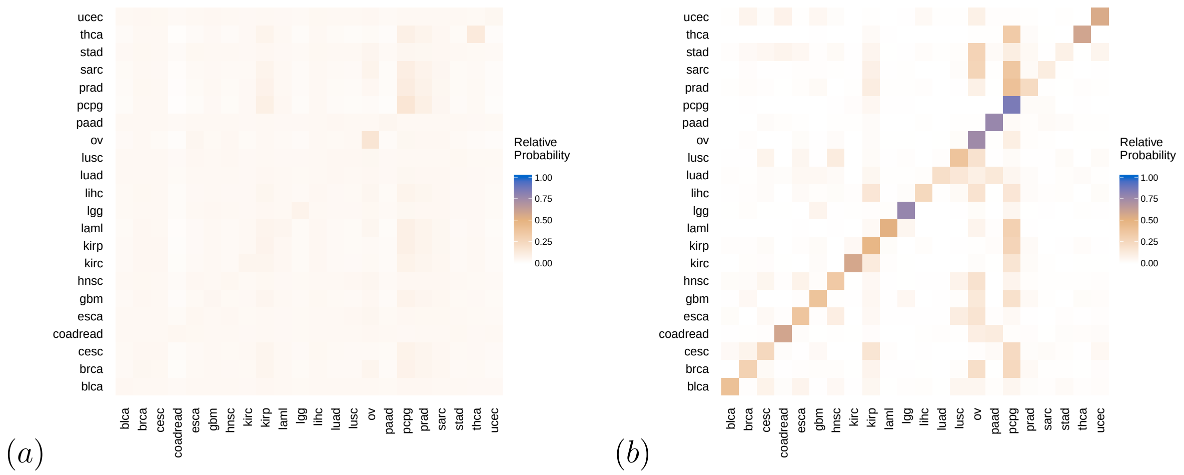


Supplementary Figure 2: Euclidean distances are used to find the centres of the cancer types, and the assignment of patient samples to each. When assigned probabilistically in (a) the average diagonal value is 7.0%. Assigning to the closest centre in (b) leads to an average of 44.4% along the diagonal.

## C Improvement of Bayesian network based cancer profiling over simple distance based approaches

For completeness we evaluate here how our Bayesian network approach to modelling mutational interactions in cancer compares to a simple distance based measure. For each cancer type we set the mean of all its mutation vectors to be the centre. Then we find the Euclidean distance between all the samples and the 22 centres. When samples are assigned to cancer types based on probabilities proportional to the inverse squared distance, the proportion of samples assigned to their own clinical cancer type is only 7.0%. Assigning samples deterministically to the closest centre instead leads to 44.4% being assigned to their nominal cancer type. Heatmaps are presented in Supplementary Figure 2.

Using a Gaussian model, still based on Euclidean distances but with the covariance structure of the mutations, gives 54.1% with a probabilistic assignment and 55.6% with a hard assignment (Supplementary Figure 3).

Using Bayesian networks instead leads to 65.5% along the diagonal when assigning probabilistically and 71.9% when assigning to the closest centre (Supplementary Figure 4).

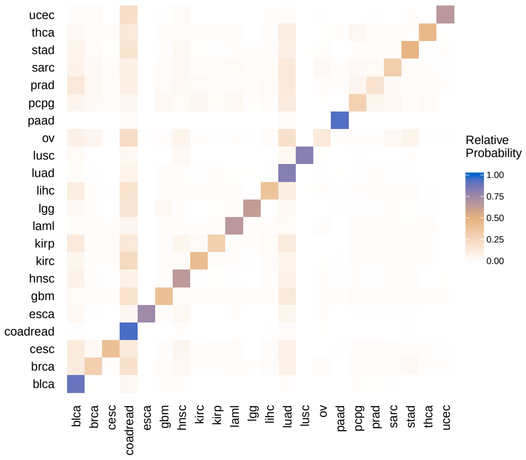


(a)

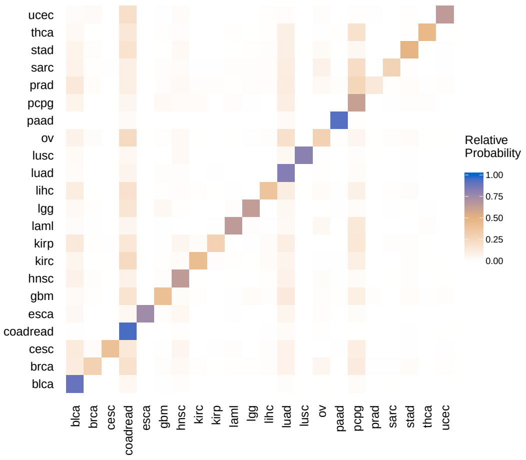


(b)

Supplementary Figure 3: Gaussian distributions using Euclidean distances are used to define the centres of the cancer types, and compute the assignment of patient samples to each. When assigned probabilistically in (a) the average diagonal value is 54.1%. Assigning to the closest centre in (b) leads to an average of 55.6% along the diagonal.

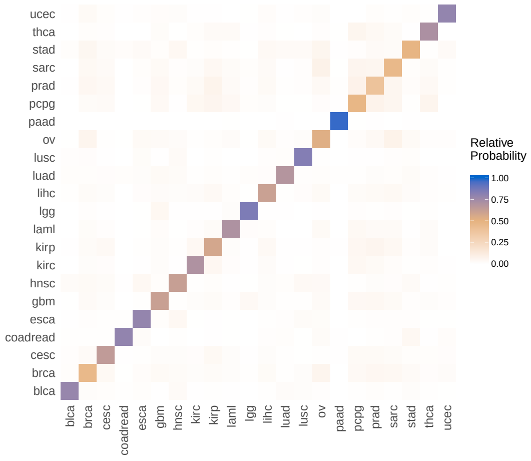


(a)

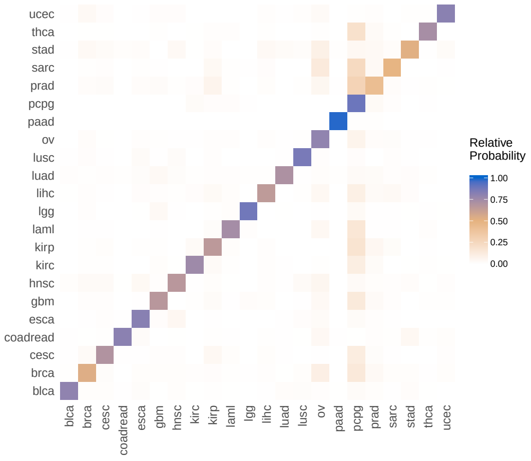


(b)

Supplementary Figure 4: A network is inferred to define the centres of the cancer types and Bayesian network based distances are employed for the assignment of patient samples to each. The probabilistic assignment in (a) results in an average of 65.5% along the diagonal. Choosing the closest centre as in (b) increases the average diagonal value to 71.9%.

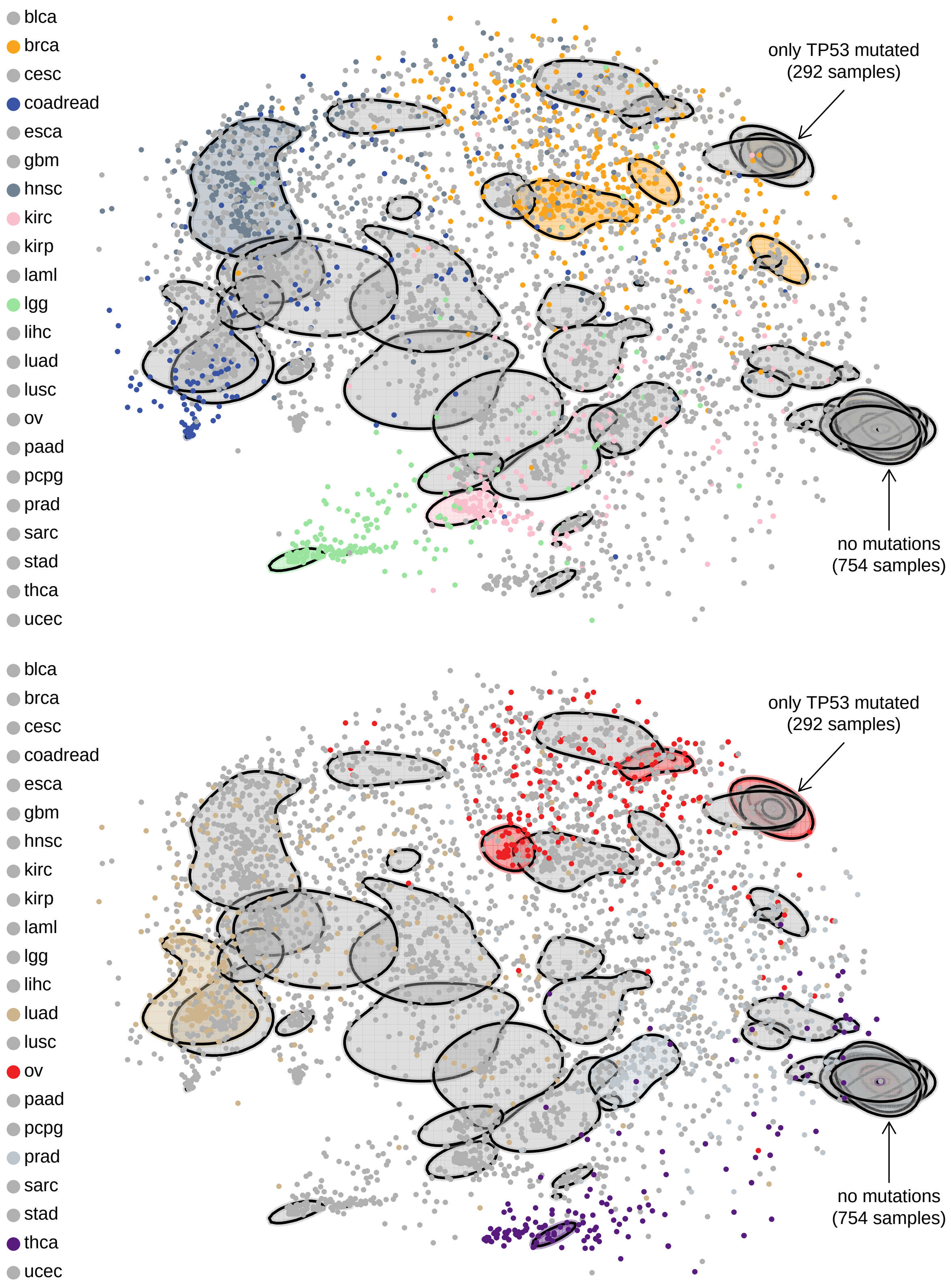


Supplementary Figure 5: Versions of Figure 3 of the main text where cancer types with more than 400 patient samples are highlighted.

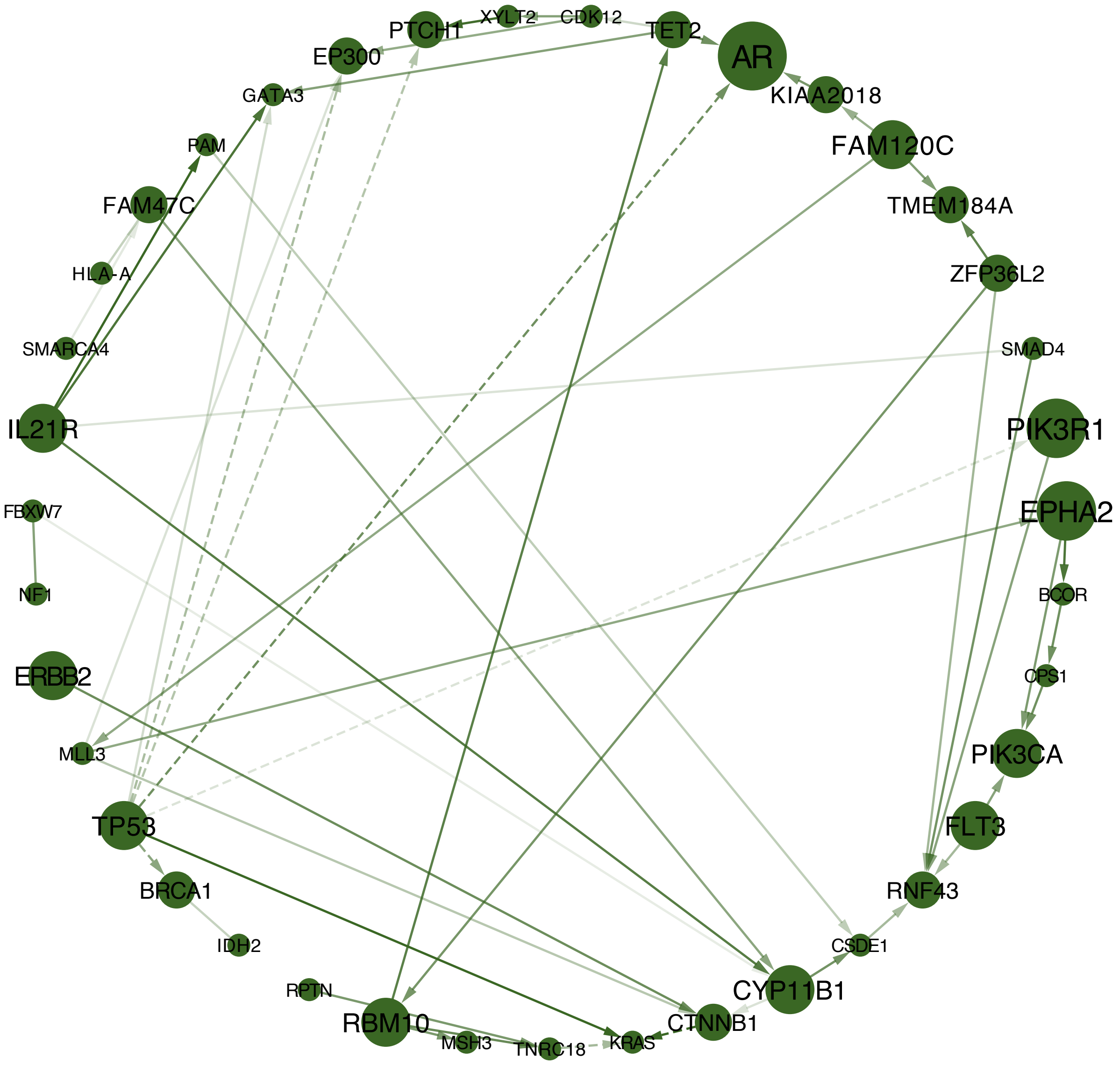


Supplementary Figure 6: For pancreatic adenocarcinoma, which is the cancer type with the fewest samples at 126, we display the posterior edge certainty from the DAG sample as the edge opacity in the network. Only genes with more than one edge with a weight above 0.5 along with edges with a weight above 0.1 are shown.

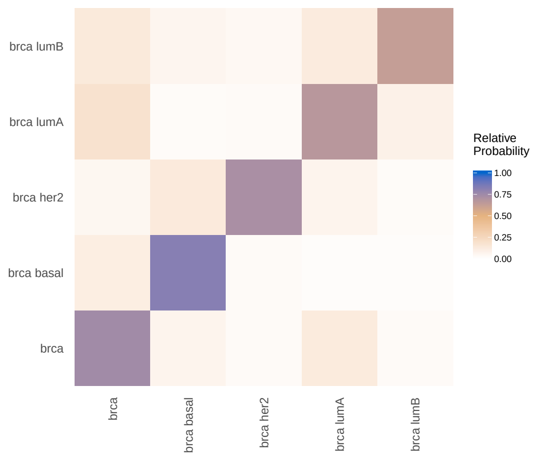


Supplementary Figure 7: A network is inferred for each breast cancer subtype and the mutation profiles of all breast cancer patient samples are assigned to the closest fitting network. The confusion matrix of the subtype labels (rows) and closest assigned network (columns) has an average diagonal value of 71.5%.

## D Breast cancer subtypes

As well as performing pancancer analyses, we can focus on individual tissue types and subtypes. Here we perform a supervised analysis only on the breast cancer samples stratified by their known subtypes (basal-like, Her2 enriched, luminal A, luminal B), for which we also use the subtype calls of [23]. For each subtype we infer Bayesian networks, which allows us to see how well the different subtypes can be distinguished (Supplementary Figure 7) and to visualise how well each patient sample fits to the different subtype-specific networks (Supplementary Figure 8). We observe a good separation of the four main subtypes, with basal being especially distinct and more overlap between the luminal subtypes, particularly luminal B, around a core of patient samples without a given subtype. This separation is in line with the assignment of patient samples to the best fitting Bayesian network (Supplementary Figure 7) with a strong self-assignment of the basal subtype and more cross-assignment for the luminal patient samples.

In the networks themselves, when we select 20 genes per subtype to display, we observe reasonably disparate modules for the different subtypes, as well as some genes with interactions across the different breast cancer subtypes (Supplementary Figure 9).

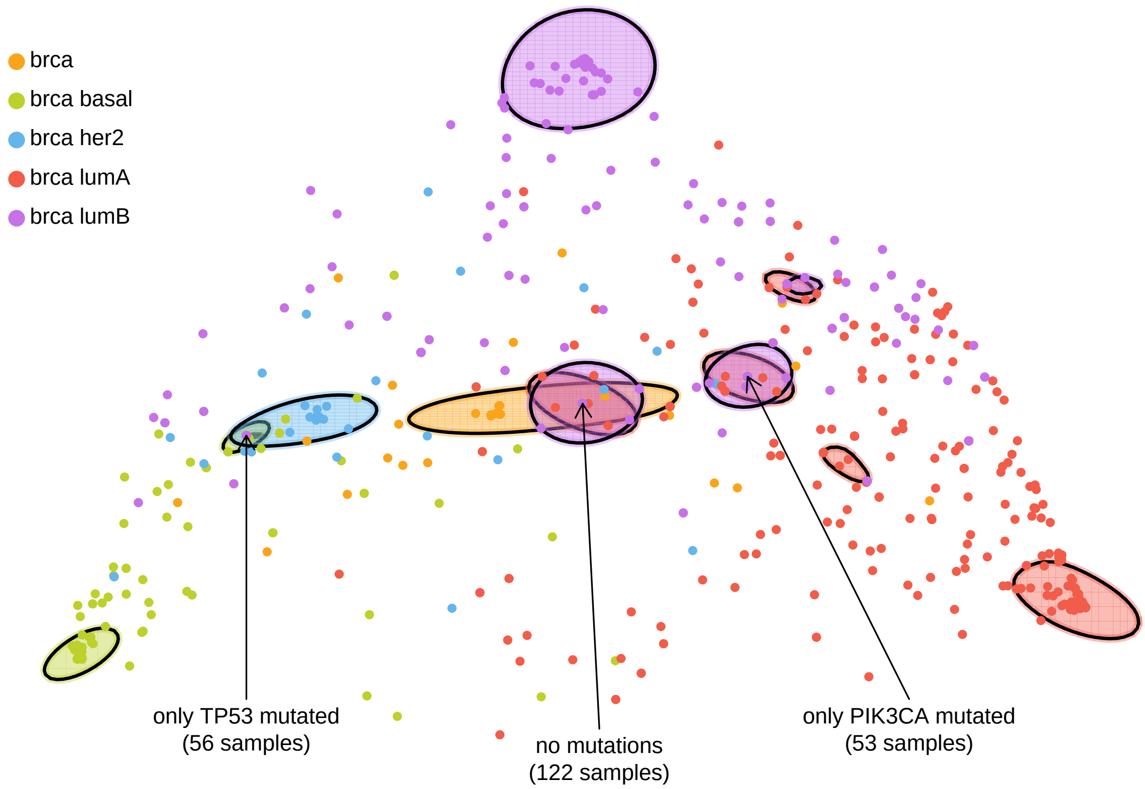



Supplementary Figure 8: 2D visualisation of the similarity between breast cancer patient samples based on Bayesian networks inferred for each known subtype. The solid contours contain a total of 50% of the respective subtype samples. Samples with no mutations, or mutations only in *TP53* of *PIK3CA* are indicated.

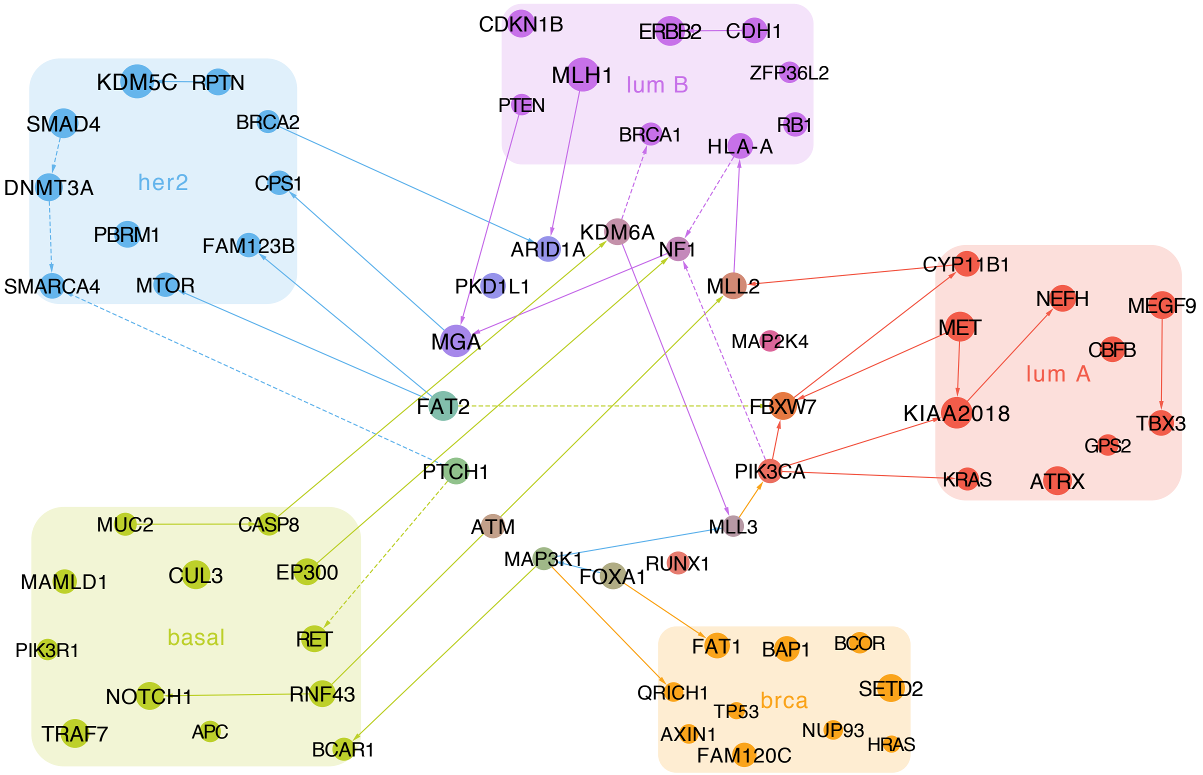


Supplementary Figure 9: The subtype-specific connections between the 20 most frequent and connected genes per subtype. Edges are coloured according to the subtype-network where they occur, if directly between the 20 selected genes per subtype, while genes are coloured according to their edges. The size of each node is proportional to the total number of edges that gene has across all subtypes, including edges not visualised.

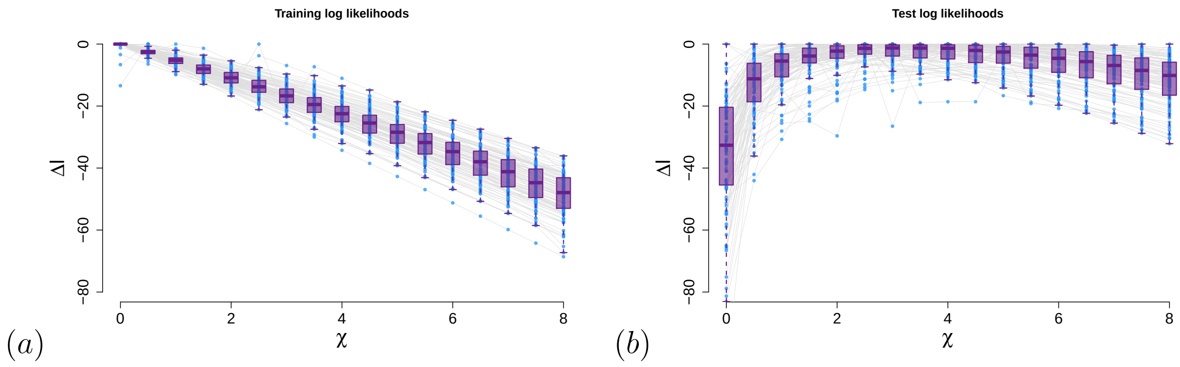


Supplementary Figure 10: Binary clustering as the prior pseudo counts $\chi$ are increased for simulated data. (a) the log-likelihood of the clusters found for the data used to cluster. (b) the likelihood of unseen data. In both cases the largest likelihood over the 100 runs is subtracted.

## E Binary clustering with a prior

Each Bayesian network model reduces to a set of independent Bernoulli variables when the networks have no edges. The Bayesian network based clustering reduces then to a standard Bernoulli mixture model. Maximum likelihood estimates of the cluster probabilities and mutation frequencies can be obtained via the EM algorithm (Methods, main text). The BDe score however includes priors on the parameters of each node, turning maximum likelihood (ML) into maximum a posteriori (MAP) clustering.

**E.1 Improvement in predictive power** To assess the effect of a prior we ran a simple simulation for $n = 16$ variables and 5 clusters consisting of $\{8, 16, 32, 64, 128\}$ samples respectively. The parameters (probabilities of each variable being 1) for each cluster were drawn uniformly.

For 100 simulated datasets we computed the likelihood of the final clustering as the prior parameter $\chi$ was varied. As expected, increasing $\chi$ reduces the fit to the data and the log-likelihoods decrease (Supplementary Figure 10a). However larger values of $\chi$ will also reduce overfitting, allowing the clustering to better predict new observations. In particular for each simulated dataset we created a partner test dataset with the same parameters as the training data. The log-likelihoods for the test data increase quickly up to $\chi \approx 2$ and then slowly fall off (Supplementary Figure 10b). The presence of a peak indicates that including a small amount of pseudocounts improves the predictive power of the binary clustering.

**E.2 Improvement in discovering the correct number of clusters** To select the number of clusters, the standard approach consists of testing the clustering for a range of possible numbers of clusters $K$ and choosing the optimal value according to some metric. For the Bernoulli mixture model, we have access to the ML estimate through the EM procedure. The natural extension is to embellish the likelihood with an information based penalty for the number of clusters, leading to metrics such as the AIC and BIC. We focus on the AIC, since it performed much better than the BIC on simulated data.

When we include the prior on the Bernoulli probabilities we can no longer directly use the

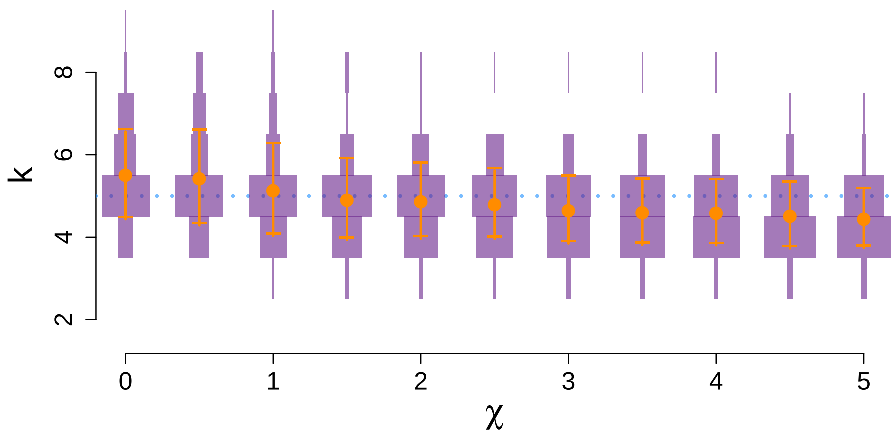


Supplementary Figure 11: The number of clusters predicted by the AIC as $\chi$ is varied for 100 simulated datasets with 5 clusters. The purple histograms depict the distribution of the number of clusters while the overlaid orange dots are the mean number of clusters with standard deviations indicated by the bars.

AIC since the prior acts as a further likelihood penalisation to reduce overfitting. In principle the AIC would need to be modified to account for this, but we take a simpler approach of first learning the clustering with pseudocounts according to $\chi$. Given the final membership probabilities, we then calculate model parameters and the likelihood and AIC by taking $\chi \to 0$. This still over penalises having $\chi \neq 0$ but less than using the AIC formula blindly. Nonetheless, having a small amount of pseudocounts improves the selection of the correct number of clusters, bringing the mean closer to the correct value of 5 and reducing the variance (Supplementary Figure 11). As $\chi$ continues to be increased, clusters are further penalised and the (modified) AIC starts to underestimate the number of clusters.

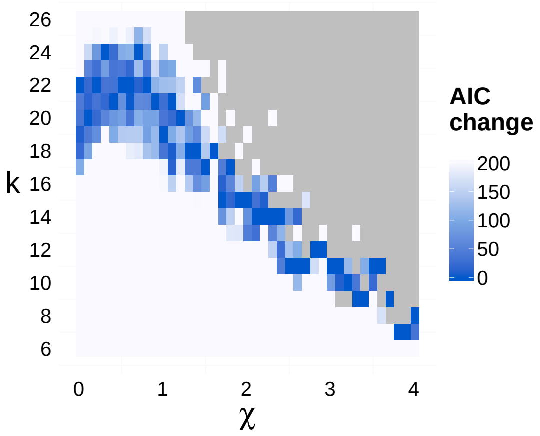


Supplementary Figure 12: The number of clusters predicted by the AIC as $\chi$ is varied for the TGCA mutation data. Grey blocks indicate the presence of empty clusters.

## F Clustering the TCGA mutation data

When we apply the Bernoulli mixture model clustering to the TCGA mutation data of the main text we vary the number of clusters $K$ and the parameter $\chi$. We cannot directly compare the modified AIC values for different values of $\chi$, since the pseudocounts themselves induce additional penalisation. Instead we simply subtract the minimum over $K$ for each value of $\chi$ separately. The resulting plot shows that we find some uncertainty in the exact number of clusters, but that having 22 clusters scores well over a range of low $\chi$ values (Supplementary Figure 12). Because a non-zero value of $\chi$ improves predictive power but too large values underestimate the number of clusters, we focus on smaller values of $\chi$. For each value of $\chi$ we take the clustering solution with the best AIC and then compare the different clusterings over $\chi$. We select the clustering with the highest similarity (normalised mutual information) to all other clusterings, leading to the value $\chi = 0.5$.

**F.1 Robustness of the clustering** To check how the parameter $\chi$ affects the clustering robustness, we can trace how the patient samples are clustered as $\chi$ is varied (Supplementary Figure 13). Samples are coloured according to their membership of the 22 clusters found for $\chi = 0.5$. Overall, patient samples tend to stay together throughout the different possible clusterings as indicated by the coloured blocks being quite stable.

To further explore the robustness of the clustering, we created 10 bootstrap samples and clustered them into 22 clusters with $\chi = 0.5$. For each bootstrap sample, we assigned the original data to the bootstrap clusters and stored the cluster labels assigned to the original data. These cluster labels were compared to those of the original clustering of the TCGA mutation data ($\chi = 0.5$ in Supplementary Figure 13). The average Rand index across the bootstrap samples was 93.1% (with a standard deviation of 0.9%) indicating a high degree of clustering robustness.

**F.2 Bayesian network clustering of the patient samples** From the starting point obtained from the Bernoulli mixture model, we now perform full clustering by learning a network model for each cluster and updating the membership probabilities until convergence. In the

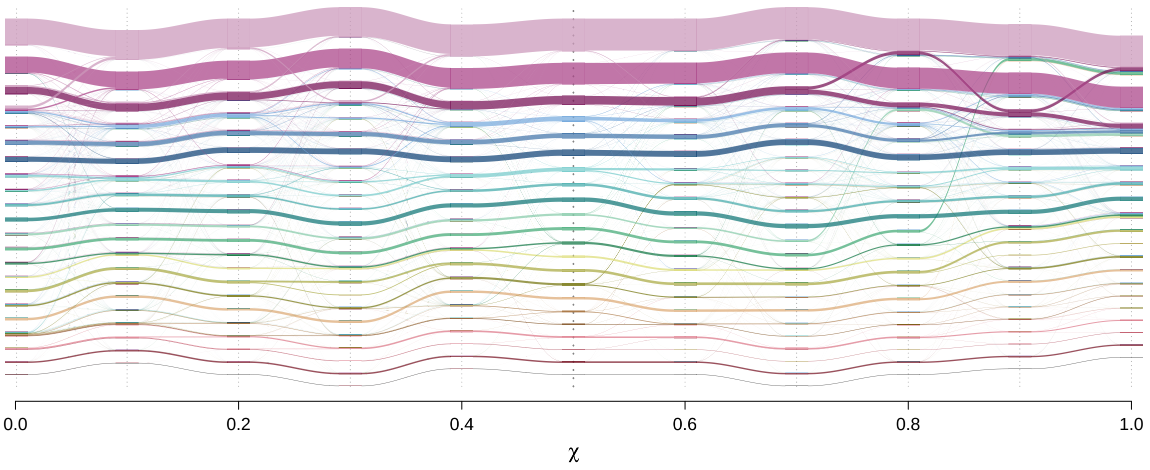


Supplementary Figure 13: Using Bernoulli mixture model clustering to reassign the patient samples we find 22 clusters around $\chi = 0.5$. Colouring the patient samples according to their cluster membership at this value, we vary the parameter $\chi$ and trace each patient sample through the differing clusters found.

network learning, edges outside the STRING network are penalised by a factor 2 (Methods). For the final assignment from the full Bayesian network clustering we can display how each patient sample from the different known cancer types and subtypes is assigned to the 22 clusters (Supplementary Figure 14) and the composition of the clusters (Supplementary Table 2).

For each Bayesian network at the heart of the 22 clusters, we can evaluate the fit for all the patient samples. To visualise how the patient samples separate out we can project into 2D (Methods, main text) and plot the resulting projection (Supplementary Figures 15 and 16).

The Bayesian networks with mutational interactions explain the data in each cluster better than just considering mutational frequencies and treating the mutations as independent with the Bernoulli mixture model. For example, the average assignment weight for each cluster of its samples increases from 88.4% to 91.5% when we take interactions into account (Supplementary Figure 17).

**F.3 Survival analysis** Kaplan-Meier curves of the survival time from diagnosis (Figure 4 of the main text) show some degree of variability in overall survival between clusters. Hazard ratios with their confidence intervals in Supplementary Table 3 show that almost all clusters have significantly different prognoses from the reference group (the largest cluster, V).

The analysis reported in Supplementary Table 3 is not adjusted for age, stage and tissue type since only the mutational data is provided to the clustering. The statistically significant differences in hazards show that the clustering is picking up biological signals in the mutational data. The unadjusted differences of course may be confounded by age, stage and tissue type. When we adjust for these covariates, clusters G, J, N and U show statistically significant differences from the reference group (Supplementary Table 4). Overall, the clusters significantly improve survival prediction (likelihood ratio $= 37$; p-value $= 7.6 \times 10^{-8}$), even when accounting for age, stage and tissue type.

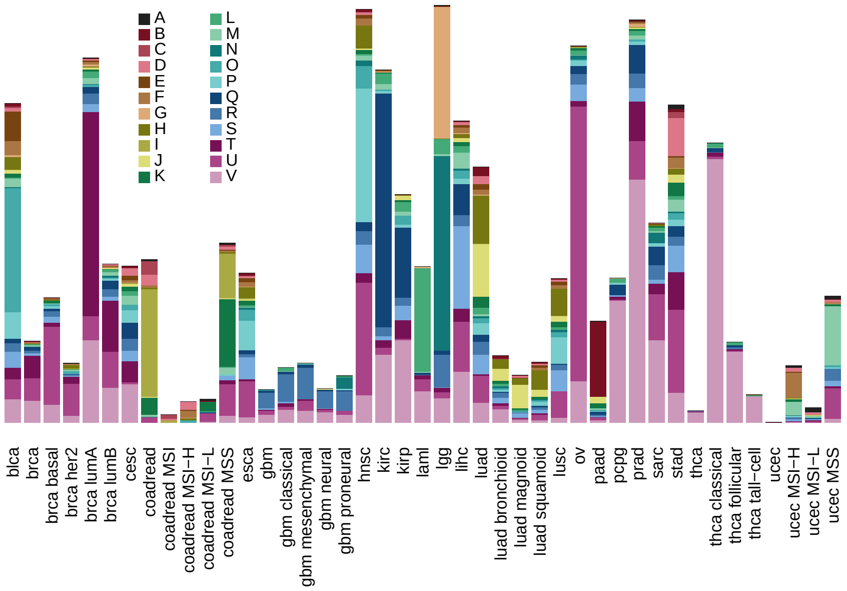


Supplementary Figure 14: Distribution of patient samples in the 22 final clusters based on the full Bayesian network modelling over cancer subtypes. The corresponding composition of the clusters is depicted in Figure 4 of the main text.

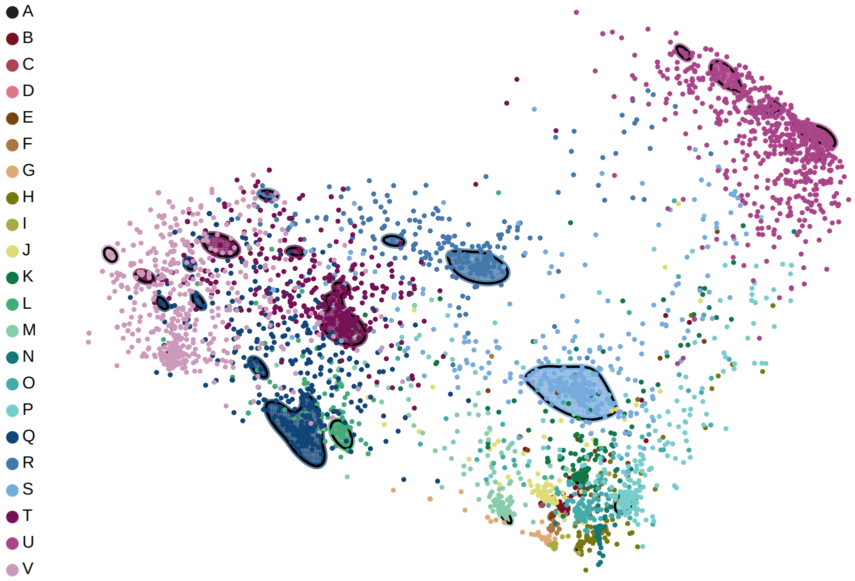


Supplementary Figure 15: The differences between the patient samples based on their fit against the Bayesian networks defining the 22 clusters are projected into 2D. The patient samples are coloured according to the cluster membership. The solid shapes together contain 50% of the probability density of each cluster.

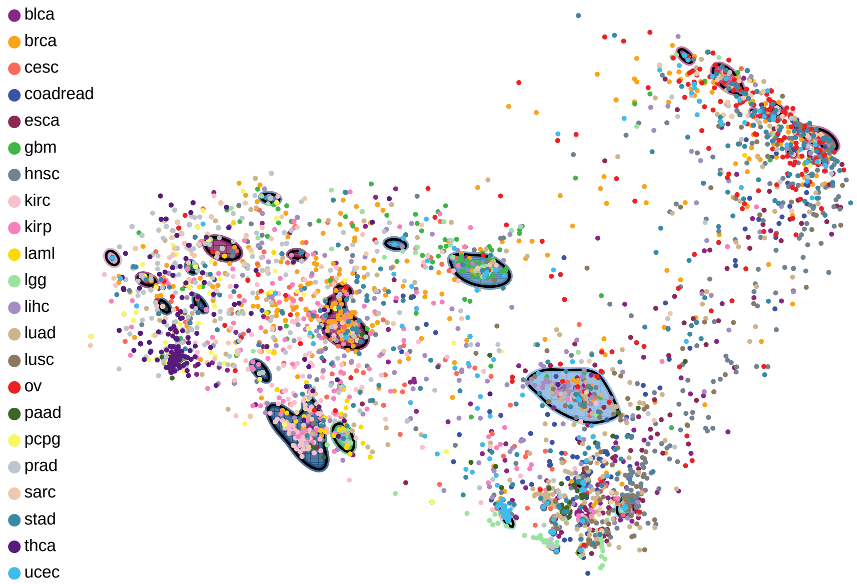


Supplementary Figure 16: The plot of Supplementary Figure 15 with the patient samples coloured by cancer type.

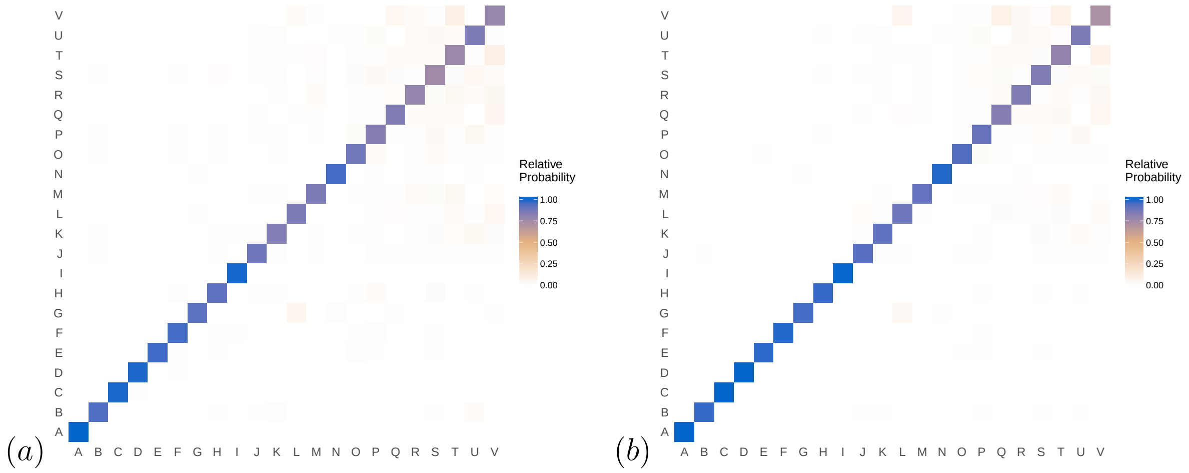


Supplementary Figure 17: When the TCGA mutation data is clustered based just on mutational frequencies, (a), the average weight along the diagonal from the probabilistic assignment of each sample to its cluster is 88.4%. When using the full Bayesian network based assignment, (b), this increases to 91.5%.

## Supplementary Table 2: Composition of the clusters

| A (29 samples) | | B (135 samples) | | C (35 samples) | | D (132 samples) | | E (84 samples) | | F (128 samples) | |
|---|---|---|---|---|---|---|---|---|---|---|---|
| ucec | 45% | paad | 69% | coadread | 69% | stad | 36% | blca | 44% | ucec | 26% |
| coadread | 21% | luad | 13% | stad | 20% | coadread | 22% | luad | 13% | blca | 13% |
| stad | 17% | blca | 3% | blca | 6% | luad | 11% | cesc | 7% | coadread | 11% |
| brca | 7% | stad | 3% | esca | 3% | ucec | 8% | esca | 6% | stad | 10% |
| lgg | 3% | cesc | 2% | hnsc | 3% | cesc | 7% | hnsc | 5% | brca | 8% |
| paad | 3% | esca | 2% | brca | 4% | lusc | 5% | hnsc | 7% | | |
| prad | 3% | hnsc | 2% | blca | 3% | brca | 4% | luad | 7% | | |
| brca | 1% | lihc | 3% | lihc | 4% | lihc | 5% | | | | |
| coadread | 1% | hnsc | 2% | coadread | 2% | esca | 5% | | | | |
| lihc | 1% | lusc | 2% | stad | 2% | lusc | 3% | | | | |

| G (181 samples) | | H (226 samples) | | I (194 samples) | | J (177 samples) | | K (222 samples) | | L (257 samples) | |
|---|---|---|---|---|---|---|---|---|---|---|---|
| lgg | 90% | luad | 46% | coadread | 98% | luad | 67% | coadread | 53% | laml | 50% |
| brca | 2% | lusc | 15% | blca | 1% | stad | 6% | luad | 10% | lgg | 7% |
| prad | 2% | hnsc | 12% | esca | 1% | paad | 5% | stad | 8% | brca | 5% |
| blca | 2% | blca | 7% | ucec | 1% | lusc | 4% | brca | 3% | kirc | 5% |
| laml | 1% | esca | 6% | kirp | 3% | lusc | 3% | kirp | 5% | | |
| kirc | 1% | brca | 3% | lihc | 3% | esca | 3% | luad | 5% | | |
| lihc | 1% | coadread | 3% | blca | 2% | paad | 3% | thca | 4% | | |
| luad | 1% | stad | 3% | brca | 2% | ucec | 3% | lihc | 3% | | |
| pcpg | 1% | lihc | 2% | cesc | 2% | blca | 2% | cesc | 2% | | |
| stad | 1% | sarc | 1% | coadread | 1% | cesc | 2% | ov | 2% | | |

| M (213 samples) | | N (302 samples) | | O (272 samples) | | P (357 samples) | | Q (625 samples) | | R (409 samples) | |
|---|---|---|---|---|---|---|---|---|---|---|---|
| ucec | 44% | lgg | 80% | blca | 56% | hnsc | 46% | kirc | 46% | gbm | 33% |
| lihc | 9% | gbm | 5% | hnsc | 10% | esca | 10% | kirp | 14% | lgg | 10% |
| brca | 7% | sarc | 4% | esca | 5% | blca | 9% | lihc | 6% | brca | 8% |
| stad | 7% | hnsc | 2% | kirp | 4% | lusc | 9% | prad | 6% | luad | 6% |
| blca | 5% | luad | 2% | brca | 4% | luad | 6% | brca | 4% | ucec | 5% |
| cesc | 5% | ov | 2% | lihc | 4% | cesc | 4% | sarc | 4% | prad | 4% |
| coadread | 5% | brca | 1% | luad | 4% | stad | 2% | cesc | 3% | sarc | 4% |
| kirc | 3% | lusc | 1% | stad | 3% | lihc | 2% | gbm | 2% | hnsc | 4% |
| hnsc | 3% | esca | 1% | cesc | 3% | ov | 2% | pcpg | 2% | cesc | 4% |
| kirp | 2% | lihc | 1% | lusc | 2% | brca | 1% | stad | 2% | lihc | 3% |

| S (413 samples) | | T (623 samples) | | U (1305 samples) | | V (1879 samples) | |
|---|---|---|---|---|---|---|---|
| lihc | 25% | brca | 57% | ov | 26% | thca | 24% |
| luad | 10% | prad | 8% | brca | 18% | prad | 16% |
| hnsc | 8% | stad | 7% | hnsc | 11% | brca | 11% |
| stad | 8% | cesc | 4% | stad | 8% | pcpg | 8% |
| esca | 7% | kirp | 4% | lihc | 5% | kirp | 5% |
| brca | 6% | lihc | 3% | coadread | 4% | sarc | 5% |
| lusc | 6% | blca | 2% | sarc | 4% | kirc | 4% |
| blca | 5% | sarc | 2% | prad | 4% | gbm | 3% |
| ov | 5% | hnsc | 2% | luad | 4% | lihc | 3% |
| kirp | 4% | kirc | 1% | esca | 3% | ov | 3% |

Supplementary Table 3: Hazard of death for each cluster relative to the reference group (cluster V), without adjustment for stage, age, or tissue type. Clusters show statistically significant differences in hazard compared to the reference group. Significant results (based on the false discovery rate, FDR $< 0.05$) are highlighted.

| Cluster | Hazard ratio | Confidence interval | | FDR |
|---|---|---|---|---|
| | | Lower | Upper | |
| A | 0.675 | 0.252 | 1.810 | 0.46 |
| B | 4.454 | 3.384 | 5.862 | $< 10^{-16}$ |
| C | 1.686 | 0.836 | 3.402 | 0.16 |
| D | 1.556 | 1.057 | 2.290 | $3.5\times10^{-2}$ |
| E | 2.462 | 1.702 | 3.561 | $2.7\times10^{-6}$ |
| F | 2.559 | 1.842 | 3.557 | $3.8\times10^{-8}$ |
| G | 0.854 | 0.564 | 1.294 | 0.46 |
| H | 2.475 | 1.916 | 3.197 | $9.4\times10^{-12}$ |
| I | 1.415 | 1.032 | 1.940 | $4.1\times10^{-2}$ |
| J | 3.540 | 2.751 | 4.554 | $< 10^{-16}$ |
| K | 2.316 | 1.750 | 3.065 | $9.1\times10^{-9}$ |
| L | 3.548 | 2.862 | 4.399 | $< 10^{-16}$ |
| M | 1.303 | 0.954 | 1.781 | 0.11 |
| N | 1.593 | 1.246 | 2.036 | $3.0\times10^{-4}$ |
| O | 2.878 | 2.300 | 3.602 | $< 10^{-16}$ |
| P | 3.308 | 2.739 | 3.995 | $< 10^{-16}$ |
| Q | 1.702 | 1.421 | 2.039 | $1.5\times10^{-8}$ |
| R | 3.698 | 3.085 | 4.432 | $< 10^{-16}$ |
| S | 2.286 | 1.863 | 2.804 | $5.8\times10^{-15}$ |
| T | 1.213 | 0.989 | 1.487 | $7.8\times10^{-2}$ |
| U | 2.513 | 2.184 | 2.892 | $< 10^{-16}$ |

Supplementary Table 4: Stage-, age-, and tissue-adjusted hazard ratio for each cluster versus the reference group (Stage I for stage and cluster V for the clusters). Stage X combines all other stage categories. Significant results are highlighted (FDR $< 0.05$).

| Cluster | Hazard ratio | Confidence interval | | FDR |
|---|---|---|---|---|
| | | Lower | Upper | |
| Stage II | 1.656 | 1.392 | 1.969 | $5.1\times10^{-8}$ |
| Stage III | 2.708 | 2.295 | 3.197 | $< 10^{-16}$ |
| Stage IV | 5.147 | 4.270 | 6.204 | $< 10^{-16}$ |
| Stage X | 2.994 | 2.325 | 3.856 | $< 10^{-16}$ |
| Age (per year increase) | 1.028 | 1.024 | 1.032 | $< 10^{-16}$ |
| A | 0.484 | 0.178 | 1.313 | 0.21 |
| B | 1.394 | 0.952 | 2.044 | 0.13 |
| C | 0.794 | 0.384 | 1.643 | 0.64 |
| D | 0.697 | 0.466 | 1.043 | 0.12 |
| E | 0.983 | 0.666 | 1.452 | 0.95 |
| F | 1.363 | 0.966 | 1.925 | 0.12 |
| G | 0.273 | 0.168 | 0.443 | $5.3\times10^{-7}$ |
| H | 0.962 | 0.728 | 1.270 | 0.84 |
| I | 0.919 | 0.595 | 1.420 | 0.81 |
| J | 1.506 | 1.132 | 2.003 | $1.0\times10^{-2}$ |
| K | 1.133 | 0.832 | 1.542 | 0.53 |
| L | 1.067 | 0.828 | 1.374 | 0.73 |
| M | 1.051 | 0.756 | 1.461 | 0.84 |
| N | 0.565 | 0.410 | 0.779 | $1.2\times10^{-3}$ |
| O | 0.893 | 0.686 | 1.164 | 0.51 |
| P | 1.194 | 0.959 | 1.486 | 0.16 |
| Q | 1.097 | 0.892 | 1.349 | 0.50 |
| R | 1.000 | 0.817 | 1.225 | 1.00 |
| S | 1.028 | 0.827 | 1.278 | 0.84 |
| T | 1.145 | 0.921 | 1.423 | 0.30 |
| U | 1.225 | 1.043 | 1.438 | $2.5\times10^{-2}$ |

Supplementary Table 5: Number of patient samples within each stage. Stage X combines all other stage categories.

| Stage | I | II | III | IV | X |
|---|---|---|---|---|---|
| Number of samples | 1544 | 1568 | 1250 | 700 | 3023 |

Supplementary Table 6: Comparison of survival prediction of different unsupervised clustering methods, without and with correction for confounding factors stage, age and tissue type. The methods with the highest predictive score are highlighted.

| Method | Uncorrected | | Corrected | |
|---|---|---|---|---|
| | LR | P-value | LR | P-value |
| Hierarchical clustering (Hamming distance) | 11.4 | 0.35 | 5.7 | 0.95 |
| Non-negative matrix factorisation | 104.7 | $5.1 \times 10^{-33}$ | 12.7 | 0.23 |
| K-means | 172.0 | $3.3 \times 10^{-60}$ | 29.8 | $1.5 \times 10^{-5}$ |
| Gaussian mixture model (mclust) | 205.6 | $4.5 \times 10^{-74}$ | 33.1 | $1.4 \times 10^{-6}$ |
| Bernoulli mixture model (no edges, $\chi = 0$) | 240.9 | $9.3 \times 10^{-89}$ | 34.0 | $7.5 \times 10^{-7}$ |
| Bernoulli mixture model (no edges, $\chi = 0.5$) | 242.4 | $2.1 \times 10^{-89}$ | 35.7 | $2.1 \times 10^{-7}$ |
| Bayesian network clustering ($\chi = 0.5$) | 253.0 | $8.0 \times 10^{-94}$ | 37.0 | $7.6 \times 10^{-8}$ |

We can also compare the Bayesian network clustering to other clustering algorithms (Supplementary Table 6) in terms of survival predictive power. We fix the number of clusters to the 22 discovered with our methods. Employing just the mutational data, and not accounting for clinical information, the Bayesian network clustering is the most informative for predicting survival. Some of the signal picked up by the clustering correlates with clinical information. When the Cox regression is adjusted for age, stage and tissue type, the Bayesian network clustering still has the best performance but the Bernoulli mixture model with a prior still performs very well with only a slightly lower LR. The Bernoulli mixture model with a prior on mutation probabilities corresponds to the special case of a network without edges. Removing the prior as well ($\chi \to 0$) leads to the classical maximum likelihood Bernoulli mixture model.

Supplementary Table 7: Mutation frequency of the 20 most frequent and connected genes per cancer type depicted in Figure 2 of the main text.

| blca | | brca | | cesc | | coadread | | esca | |
|---|---|---|---|---|---|---|---|---|---|
| TP53 | 0.496 | TP53 | 0.305 | MLL3 | 0.155 | PTEN | 0.452 | TP53 | 0.827 |
| KDM6A | 0.271 | PIK3CA | 0.325 | EP300 | 0.113 | PIK3CA | 0.495 | MLL2 | 0.184 |
| STAG2 | 0.157 | CDH1 | 0.111 | FAT2 | 0.067 | ATM | 0.37 | ATM | 0.119 |
| ERBB2 | 0.144 | MAP3K1 | 0.074 | MTOR | 0.067 | TP53 | 0.687 | KIAA2018 | 0.076 |
| RB1 | 0.177 | GATA3 | 0.101 | MLL2 | 0.144 | KIT | 0.274 | EP300 | 0.07 |
| MLL2 | 0.301 | MLL2 | 0.028 | ARID1A | 0.077 | ARID1A | 0.2 | PIK3CA | 0.103 |
| FGFR3 | 0.144 | NF1 | 0.031 | HLA-B | 0.057 | MLL2 | 0.155 | MTOR | 0.059 |
| ATM | 0.144 | ARID1A | 0.03 | AR | 0.052 | RNF43 | 0.1 | MGA | 0.086 |
| CDK12 | 0.078 | ATRX | 0.018 | NFE2L2 | 0.067 | FBXW7 | 0.325 | STK11 | 0.032 |
| ERCC2 | 0.101 | MTOR | 0.021 | RB1 | 0.046 | MLL3 | 0.162 | TSC1 | 0.054 |
| FAT2 | 0.094 | ATM | 0.025 | BRCA1 | 0.031 | PTCH1 | 0.272 | FLT3 | 0.054 |
| KDR | 0.046 | FAT1 | 0.025 | HLA-A | 0.088 | VHL | 0.213 | PTCH1 | 0.07 |
| ARID2 | 0.084 | NSD1 | 0.014 | ATM | 0.057 | RB1 | 0.258 | CDK12 | 0.027 |
| EP300 | 0.154 | PTCH1 | 0.013 | ERBB2 | 0.057 | KRAS | 0.491 | XYLT2 | 0.011 |
| KIAA2018 | 0.076 | ERCC6 | 0.017 | CASP8 | 0.046 | NF2 | 0.178 | ERBB2 | 0.059 |
| MGA | 0.071 | FAT2 | 0.024 | MAP3K1 | 0.026 | FAT1 | 0.096 | CDKN2A | 0.114 |
| LTBP3 | 0.028 | MET | 0.009 | LARP1 | 0.041 | APC | 0.847 | ATRX | 0.022 |
| FAT1 | 0.137 | SETD2 | 0.018 | NOTCH1 | 0.062 | BRAF | 0.268 | MET | 0.027 |
| DNMT3A | 0.046 | PKD1L1 | 0.023 | MUC2 | 0.057 | CTNNB1 | 0.241 | CPS1 | 0.054 |
| CDKN2A | 0.066 | LMTK2 | 0.013 | DNMT3A | 0.015 | EGFR | 0.225 | MLL3 | 0.103 |
| gbm | | hnsc | | kirc | | kirp | | laml | |
| TP53 | 0.283 | TP53 | 0.712 | MLL3 | 0.041 | SETD2 | 0.06 | FLT3 | 0.269 |
| PTEN | 0.307 | CASP8 | 0.106 | MTOR | 0.073 | AR | 0.05 | TP53 | 0.078 |
| RB1 | 0.088 | FAT1 | 0.237 | PIK3CA | 0.023 | MET | 0.074 | IDH2 | 0.104 |
| IDH1 | 0.049 | PIK3CA | 0.186 | MLL2 | 0.037 | STAG2 | 0.028 | TET2 | 0.088 |
| ATRX | 0.064 | MLL2 | 0.16 | FAT2 | 0.018 | MLL2 | 0.057 | RUNX1 | 0.083 |
| PIK3CA | 0.099 | APC | 0.045 | ATM | 0.03 | BAP1 | 0.05 | U2AF1 | 0.041 |
| BRCA1 | 0.014 | MLL3 | 0.096 | BRCA2 | 0.014 | TP53 | 0.025 | BCOR | 0.01 |
| MLL3 | 0.049 | CDKN2A | 0.219 | PLEKHA6 | 0.014 | KIAA2018 | 0.043 | EGFR | 0.01 |
| BRAF | 0.025 | CPS1 | 0.041 | MUC2 | 0.025 | PBRM1 | 0.039 | MTA2 | 0.01 |
| STAG2 | 0.042 | EGFR | 0.049 | TP53 | 0.023 | PIK3CA | 0.018 | ARID1A | 0.005 |
| BCOR | 0.035 | NOTCH1 | 0.19 | ARID1A | 0.023 | KDM6A | 0.035 | HLA-B | 0.005 |
| CPS1 | 0.018 | EPHA2 | 0.049 | NSD1 | 0.021 | CUL3 | 0.035 | FAT2 | 0.005 |
| MAP3K1 | 0.028 | SMARCA4 | 0.049 | FAT1 | 0.021 | NF1 | 0.018 | PTCH1 | 0.005 |
| SEMG1 | 0.028 | COL4A3 | 0.045 | MLH1 | 0.005 | ARID1A | 0.032 | KCNN3 | 0.005 |
| SLC28A1 | 0.014 | HRAS | 0.061 | LMTK2 | 0.009 | DPCR1 | 0.011 | ARHGAP18 | 0.005 |
| EP300 | 0.011 | NSD1 | 0.121 | TCF12 | 0.009 | ERBB2 | 0.014 | NPNT | 0.005 |
| CYP11B1 | 0.011 | RAC1 | 0.029 | EP300 | 0.016 | ERCC6 | 0.014 | DNMT3A | 0.249 |
| RET | 0.011 | PKD1L1 | 0.039 | APC | 0.016 | FGFR3 | 0.025 | NPM1 | 0.171 |
| AXIN1 | 0.011 | NF1 | 0.035 | RB1 | 0.005 | PKD2 | 0.007 | IDH1 | 0.093 |
| MLL2 | 0.018 | TGFBR2 | 0.051 | JUB | 0.007 | VHL | 0.011 | NRAS | 0.078 |
| lgg | | lihc | | luad | | lusc | | ov | |
| IDH1 | 0.777 | TP53 | 0.308 | TP53 | 0.538 | TP53 | 0.82 | TP53 | 0.826 |
| TP53 | 0.486 | CTNNB1 | 0.265 | MLL3 | 0.178 | NF1 | 0.129 | BRCA1 | 0.039 |
| ATRX | 0.386 | FAT2 | 0.062 | KRAS | 0.302 | MLL3 | 0.18 | APC | 0.028 |
| CIC | 0.209 | RB1 | 0.056 | FAT1 | 0.118 | CDKN2A | 0.152 | NF1 | 0.052 |
| NOTCH1 | 0.083 | AXIN1 | 0.067 | STK11 | 0.148 | FAM47C | 0.112 | CPS1 | 0.021 |
| FUBP1 | 0.091 | EGFR | 0.021 | CPS1 | 0.139 | NOTCH1 | 0.096 | RB1 | 0.032 |
| EGFR | 0.07 | BRCA2 | 0.032 | MLL2 | 0.107 | CYP11B1 | 0.084 | FAT2 | 0.021 |
| PIK3CA | 0.085 | MLL2 | 0.062 | FAM47C | 0.154 | PIK3CA | 0.157 | KIT | 0.021 |
| PTEN | 0.047 | ATRX | 0.024 | EGFR | 0.141 | APC | 0.051 | ERBB2 | 0.011 |
| FAM47C | 0.045 | SHROOM4 | 0.024 | ARID2 | 0.062 | CPS1 | 0.146 | MLL2 | 0.009 |
| IDH2 | 0.039 | MLL3 | 0.059 | BRCA2 | 0.06 | SETD2 | 0.028 | EGFR | 0.015 |
| FAT1 | 0.025 | MGA | 0.056 | PKD1L1 | 0.116 | ATRX | 0.067 | ARID2 | 0.015 |
| NF1 | 0.064 | CPS1 | 0.035 | PTCH1 | 0.038 | NSD1 | 0.062 | CYP11B1 | 0.015 |
| SETD2 | 0.019 | STAG2 | 0.032 | ATRX | 0.092 | FTSJD1 | 0.028 | EFEMP1 | 0.015 |
| SMARCA4 | 0.052 | TBX3 | 0.019 | KEAP1 | 0.174 | TBX3 | 0.056 | PKD1L1 | 0.015 |
| DNMT3A | 0.023 | FAT1 | 0.046 | GPR50 | 0.043 | EPHA2 | 0.017 | PIK3CA | 0.013 |
| ARID1A | 0.043 | ARID1A | 0.091 | EPAS1 | 0.032 | FLT3 | 0.051 | FBXW7 | 0.013 |
| GATA6 | 0.01 | CDC27 | 0.043 | FAM123B | 0.077 | KDR | 0.096 | PAXIP1 | 0.006 |
| BCOR | 0.039 | FLT3 | 0.013 | BRAF | 0.073 | FAT2 | 0.096 | NSD1 | 0.011 |
| FAT2 | 0.033 | TNRC18 | 0.04 | ERCC6 | 0.045 | NEFH | 0.022 | PTEN | 0.011 |
| paad | | pcpg | | prad | | sarc | | stad | |
| TP53 | 0.643 | NF1 | 0.084 | SPOP | 0.114 | TP53 | 0.344 | MLL2 | 0.183 |

*Continued on next page*

| | | | | | | | | | |
|---|---|---|---|---|---|---|---|---|---|
| KRAS | 0.794 | FAT1 | 0.017 | TP53 | 0.114 | LTBP3 | 0.02 | ARID1A | 0.252 |
| FAM47C | 0.175 | BCOR | 0.017 | MLL3 | 0.064 | RPTN | 0.016 | XYLT2 | 0.107 |
| SMAD4 | 0.206 | SETD2 | 0.017 | MLL2 | 0.06 | ATM | 0.024 | RNF43 | 0.115 |
| AR | 0.063 | NUDT11 | 0.028 | ARID1A | 0.018 | TNRC18 | 0.024 | LARP4B | 0.084 |
| RBM10 | 0.087 | BRCA2 | 0.011 | JUB | 0.004 | PIK3CA | 0.024 | MTOR | 0.084 |
| RNF43 | 0.071 | MUC2 | 0.011 | CDH1 | 0.01 | FGFR3 | 0.008 | NOTCH1 | 0.097 |
| RPTN | 0.103 | CSDE1 | 0.022 | PTEN | 0.034 | FAT1 | 0.04 | MUC2 | 0.112 |
| KIAA2018 | 0.063 | STAG2 | 0.011 | PIK3CA | 0.032 | MLL2 | 0.02 | PIK3CA | 0.165 |
| IRS1 | 0.183 | SLC26A3 | 0.011 | FAT2 | 0.032 | CYP11B1 | 0.02 | FAT1 | 0.102 |
| MLL3 | 0.087 | NOTCH1 | 0.011 | BCLAF1 | 0.016 | NF1 | 0.036 | SMARCA4 | 0.087 |
| ATRX | 0.167 | CDC27 | 0.011 | EPHA2 | 0.004 | MSH3 | 0.036 | MLL3 | 0.145 |
| PIK3CA | 0.04 | ARID2 | 0.011 | CTNNB1 | 0.026 | EGFR | 0.008 | IRS1 | 0.071 |
| CYP11B1 | 0.032 | CD99L2 | 0.011 | FAM47C | 0.026 | XYLT2 | 0.008 | APC | 0.137 |
| CTNNB1 | 0.04 | SRPX | 0.011 | EME2 | 0.006 | NUMBL | 0.032 | ATM | 0.109 |
| QRICH1 | 0.111 | TP53 | 0.006 | IDH1 | 0.012 | NUP93 | 0.008 | MGA | 0.076 |
| SHROOM4 | 0.095 | KIT | 0.006 | KEAP1 | 0.008 | LMTK2 | 0.016 | BRAF | 0.059 |
| EP300 | 0.032 | CRIPAK | 0.006 | ARID2 | 0.022 | PTEN | 0.028 | ERCC6 | 0.074 |
| TMEM184A | 0.032 | MTOR | 0.006 | BRCA2 | 0.022 | MUC2 | 0.024 | BRCA2 | 0.099 |
| EPHA2 | 0.016 | AR | 0.006 | RNF43 | 0.01 | PBRM1 | 0.012 | TP53 | 0.483 |

| thca | | ucec | |
|---|---|---|---|
| BRAF | 0.589 | PTEN | 0.649 |
| NRAS | 0.081 | PIK3CA | 0.532 |
| HRAS | 0.034 | MLL3 | 0.129 |
| DLX6 | 0.014 | FAT1 | 0.181 |
| IRS1 | 0.012 | TP53 | 0.286 |
| ABL1 | 0.012 | PIK3R1 | 0.335 |
| DNMT3A | 0.01 | MTOR | 0.113 |
| ARID2 | 0.01 | ARID1A | 0.343 |
| RBM10 | 0.01 | ATRX | 0.109 |
| NSD1 | 0.008 | APC | 0.121 |
| TNRC18 | 0.008 | SETD2 | 0.093 |
| GPR44 | 0.008 | ATM | 0.125 |
| NUP93 | 0.008 | MLL2 | 0.149 |
| SRPX | 0.008 | KDM5C | 0.073 |
| MAP3K1 | 0.006 | STAG2 | 0.101 |
| BCLAF1 | 0.006 | BRCA2 | 0.101 |
| CDK12 | 0.006 | KIT | 0.073 |
| BRCA2 | 0.006 | GPR128 | 0.065 |
| MAMLD1 | 0.006 | FBXW7 | 0.157 |
| CD99L2 | 0.006 | NF1 | 0.105 |

Supplementary Table 8: Pathway enrichment (FDR $< 0.05$) of highly connected genes across all cancer types (at least 22 edges, or one per cancer type on average) against KEGG pathways [39]. Pathways also significantly enriched in genes with $< 22$ edges across all cancer types are not shown.

| KEGG Pathway | FDR |
|---|---|
| FoxO signaling pathway | $8.2 \times 10^{-12}$ |
| PI3K-Akt signaling pathway | $8.3 \times 10^{-11}$ |
| Cellular senescence | $8.3 \times 10^{-11}$ |
| Adherens junction | $2.5 \times 10^{-10}$ |
| Rap1 signaling pathway | $3.9 \times 10^{-10}$ |
| Signaling pathways regulating pluripotency of stem cells | $6.1 \times 10^{-10}$ |
| ErbB signaling pathway | $1.3 \times 10^{-09}$ |
| Neurotrophin signaling pathway | $7.2 \times 10^{-09}$ |
| Ras signaling pathway | $1.4 \times 10^{-08}$ |
| mTOR signaling pathway | $1.4 \times 10^{-08}$ |
| Focal adhesion | $7.4 \times 10^{-08}$ |
| MAPK signaling pathway | $8.0 \times 10^{-08}$ |
| Longevity regulating pathway | $2.6 \times 10^{-07}$ |
| Thyroid hormone signaling pathway | $3.9 \times 10^{-07}$ |
| Cell cycle | $7.2 \times 10^{-07}$ |
| VEGF signaling pathway | $7.2 \times 10^{-07}$ |
| Autophagy | $9.7 \times 10^{-07}$ |
| Sphingolipid signaling pathway | $3.6 \times 10^{-06}$ |
| Phospholipase D signaling pathway | $3.6 \times 10^{-06}$ |
| Axon guidance | $3.6 \times 10^{-06}$ |
| Regulation of actin cytoskeleton | $3.9 \times 10^{-06}$ |
| HIF-1 signaling pathway | $5.5 \times 10^{-06}$ |
| Relaxin signaling pathway | $7.5 \times 10^{-06}$ |
| Natural killer cell mediated cytotoxicity | $7.8 \times 10^{-06}$ |
| Insulin signaling pathway | $1.1 \times 10^{-05}$ |
| Fc epsilon RI signaling pathway | $1.9 \times 10^{-05}$ |
| B cell receptor signaling pathway | $2.6 \times 10^{-05}$ |
| Hippo signaling pathway | $3.2 \times 10^{-05}$ |
| Melanogenesis | $4.1 \times 10^{-05}$ |
| C-type lectin receptor signaling pathway | $4.9 \times 10^{-05}$ |
| Aldosteron-regulated sodium reabsorption | $4.9 \times 10^{-05}$ |
| Apoptosis | $6.2 \times 10^{-05}$ |
| TGF-beta signaling pathway | $7.7 \times 10^{-05}$ |
| Wnt signaling pathway | $1.1 \times 10^{-04}$ |
| Long-term potentiation | $1.4 \times 10^{-04}$ |
| Chemokine signaling pathway | $1.5 \times 10^{-04}$ |
| Prolactin signaling pathway | $1.8 \times 10^{-04}$ |
| T cell receptor signaling pathway | $2.6 \times 10^{-04}$ |
| Apelin signaling pathway | $3.5 \times 10^{-04}$ |
| Estrogen signaling pathway | $3.5 \times 10^{-04}$ |
| Th17 cell differentiation | $3.6 \times 10^{-04}$ |
| Lysine degradation | $5.6 \times 10^{-04}$ |

*Continued on next page*

Supplementary Table 8 – *Continued from previous page*

| KEGG Pathway | FDR |
|---|---|
| Long-term depression | $6.0 \times 10^{-04}$ |
| GnRH signaling pathway | $9.6 \times 10^{-04}$ |
| cAMP signaling pathway | $1.1 \times 10^{-03}$ |
| Progesteron-mediated oocyte maturation | $1.4 \times 10^{-03}$ |
| p53 signaling pathway | $1.5 \times 10^{-03}$ |
| Ubiquitin mediated proteolysis | $1.7 \times 10^{-03}$ |
| Parathyroid hormone synthesis, secretion and action | $1.9 \times 10^{-03}$ |
| Leukocyte transendothelial migration | $2.6 \times 10^{-03}$ |
| Cholinergic synapse | $2.6 \times 10^{-03}$ |
| Oxytocin signaling pathway | $3.2 \times 10^{-03}$ |
| AMPK signaling pathway | $3.8 \times 10^{-03}$ |
| Gap junction | $3.9 \times 10^{-03}$ |
| Jak-STAT signaling pathway | $4.6 \times 10^{-03}$ |
| Mitophagy | $6.0 \times 10^{-03}$ |
| Tight junction | $6.1 \times 10^{-03}$ |
| Homologous recombination | $7.4 \times 10^{-03}$ |
| Toll-like receptor signaling pathway | $8.5 \times 10^{-03}$ |
| Thermogenesis | $1.0 \times 10^{-02}$ |
| Hedgehog signaling pathway | $1.2 \times 10^{-02}$ |
| Notch signaling pathway | $1.2 \times 10^{-02}$ |
| Serotonergic synapse | $1.3 \times 10^{-02}$ |
| Endocytosis | $1.5 \times 10^{-02}$ |
| Fanconi anemia pathway | $1.8 \times 10^{-02}$ |
| Regulation of lipolysis in adipocytes | $1.8 \times 10^{-02}$ |
| Osteoclast differentiation | $2.1 \times 10^{-02}$ |
| Fc gamma R-mediated phagocytosis | $2.1 \times 10^{-02}$ |
| Adipocytokine signaling pathway | $4.0 \times 10^{-02}$ |
| TNF signaling pathway | $4.2 \times 10^{-02}$ |
| D-Arginine and D-ornithine metabolism | $4.2 \times 10^{-02}$ |

Supplementary Table 9: Mutation frequency of the 20 most frequent and connected genes per cluster depicted in Figure 5 of the main text.

| A | | B | | C | | D | | E | |
|---|---|---|---|---|---|---|---|---|---|
| MTOR | 0.862 | TP53 | 0.704 | MLL2 | 0.829 | CIC | 0.326 | ARID1A | 0.393 |
| ERCC6 | 0.724 | SMAD4 | 0.237 | ATM | 0.829 | MTOR | 0.318 | TP53 | 0.738 |
| CTNNB1 | 0.621 | IRS1 | 0.185 | PIK3CA | 0.743 | PIK3CA | 0.455 | ERBB2 | 0.357 |
| BRCA2 | 0.931 | IFT46 | 0.096 | NF1 | 0.743 | XYLT2 | 0.295 | EP300 | 0.286 |
| CPS1 | 0.724 | FAM47C | 0.207 | PTCH1 | 0.686 | FAT1 | 0.439 | RB1 | 0.345 |
| EP300 | 0.69 | C19orf55 | 0.119 | FBXW7 | 0.629 | RET | 0.205 | FAM47C | 0.202 |
| CDC27 | 0.586 | RPTN | 0.111 | BRAF | 0.629 | MLL2 | 0.644 | BRCA2 | 0.179 |
| IRS1 | 0.655 | C15orf24 | 0.104 | TP53 | 0.6 | ATRX | 0.318 | FAT1 | 0.143 |
| RNF43 | 0.483 | NF1 | 0.052 | CTNNB1 | 0.486 | ARID1A | 0.614 | PAXIP1 | 0.107 |
| EPAS1 | 0.483 | BRCA2 | 0.044 | APC | 0.914 | SMARCA4 | 0.303 | EPHA2 | 0.119 |
| EPHA2 | 0.379 | SMARCA4 | 0.022 | EP300 | 0.371 | EGFR | 0.288 | SMG7 | 0.119 |
| SETD2 | 0.931 | CYP11B1 | 0.067 | VHL | 0.514 | PTCH1 | 0.288 | PKD1L1 | 0.226 |
| STAG2 | 0.724 | HLA-A | 0.03 | SMAD4 | 0.486 | ARID2 | 0.265 | ERCC2 | 0.214 |
| MSH3 | 0.552 | CIC | 0.03 | NF2 | 0.4 | PIK3R1 | 0.174 | R3HDM1 | 0.131 |
| FAM47C | 0.655 | MLL2 | 0.059 | ARID2 | 0.343 | AR | 0.227 | GATA3 | 0.071 |
| MAP3K1 | 0.793 | AR | 0.059 | KRAS | 0.457 | TP53 | 0.455 | SMARCA4 | 0.179 |
| SLC26A3 | 0.621 | KDM6A | 0.052 | FAT2 | 0.314 | FAM123B | 0.22 | STK11 | 0.06 |
| PIK3CA | 0.759 | KIAA2018 | 0.052 | NSD1 | 0.257 | ATM | 0.432 | HRAS | 0.083 |
| ATRX | 0.724 | GAGE2A | 0.052 | TNRC18 | 0.4 | BRCA1 | 0.212 | MLL2 | 0.31 |
| MET | 0.69 | STK11 | 0.052 | RBM10 | 0.286 | LMTK2 | 0.205 | ATM | 0.155 |
| F | | G | | H | | I | | J | |
| PTEN | 0.422 | IDH1 | 0.983 | TP53 | 0.925 | TP53 | 0.871 | KRAS | 0.689 |
| MLL2 | 0.359 | NOTCH1 | 0.182 | MLL3 | 0.327 | KIT | 0.495 | MLL2 | 0.09 |
| ATM | 0.211 | PIK3CA | 0.155 | PIK3CA | 0.173 | VHL | 0.423 | ATM | 0.22 |
| ARID1A | 0.258 | TP53 | 0.044 | BRCA2 | 0.173 | EGFR | 0.392 | CPS1 | 0.158 |
| BRAF | 0.164 | NF1 | 0.055 | MLL2 | 0.177 | MLH1 | 0.258 | TSC1 | 0.062 |
| BRCA2 | 0.164 | FAT1 | 0.017 | CPS1 | 0.239 | NF2 | 0.335 | MET | 0.056 |
| MGA | 0.234 | MLL3 | 0.044 | BRCA1 | 0.133 | ATM | 0.608 | KDM5C | 0.107 |
| ARID2 | 0.148 | FAM47C | 0.044 | ARID2 | 0.102 | SMAD4 | 0.577 | MGA | 0.051 |
| KRAS | 0.211 | RPTN | 0.011 | NSD1 | 0.15 | RET | 0.139 | MLL3 | 0.096 |
| MTOR | 0.203 | BRCA2 | 0.017 | ATRX | 0.142 | ERBB2 | 0.124 | SETD2 | 0.023 |
| NSD1 | 0.133 | PKD1L1 | 0.017 | RB1 | 0.093 | STK11 | 0.16 | FAT1 | 0.085 |
| FAT2 | 0.18 | CD99L2 | 0.017 | FAT1 | 0.252 | MET | 0.237 | KDR | 0.079 |
| PTCH1 | 0.18 | SHROOM4 | 0.017 | SHROOM4 | 0.124 | NOTCH1 | 0.155 | DNMT3A | 0.073 |
| FBXW7 | 0.172 | BCOR | 0.028 | ERCC6 | 0.097 | RB1 | 0.464 | WT1 | 0.034 |
| EP300 | 0.156 | SMARCB1 | 0.011 | PKD1L1 | 0.19 | PTCH1 | 0.459 | CTNNB1 | 0.034 |
| RHOA | 0.102 | TMEM184A | 0.011 | LARP4B | 0.062 | PIK3R1 | 0.206 | FAT2 | 0.068 |
| CIC | 0.141 | CTNNB1 | 0.022 | SLC26A3 | 0.053 | BRCA1 | 0.175 | EOMES | 0.028 |
| FAM123B | 0.141 | CDK12 | 0.022 | MET | 0.053 | CDH1 | 0.34 | AR | 0.028 |
| NOTCH1 | 0.141 | GPR50 | 0.011 | CYP11B1 | 0.155 | SOX9 | 0.113 | TGFBR2 | 0.051 |
| MLL3 | 0.25 | MGA | 0.011 | MUC2 | 0.15 | ABL1 | 0.103 | BCLAF1 | 0.051 |
| K | | L | | M | | N | | O | |
| APC | 0.743 | FLT3 | 0.249 | CTNNB1 | 0.427 | IDH1 | 0.858 | TP53 | 0.511 |
| TP53 | 0.568 | IDH2 | 0.16 | PIK3CA | 0.535 | TP53 | 0.977 | ARID1A | 0.25 |
| PTEN | 0.099 | NPM1 | 0.14 | KRAS | 0.225 | FAM47C | 0.043 | FGFR3 | 0.188 |
| ATM | 0.072 | DNMT3A | 0.249 | PIK3R1 | 0.183 | AR | 0.02 | RB1 | 0.136 |
| MLL3 | 0.059 | MLL2 | 0.043 | ERBB2 | 0.056 | EGFR | 0.017 | MLL2 | 0.257 |
| FAT2 | 0.113 | RUNX1 | 0.089 | MTOR | 0.042 | NOTCH1 | 0.033 | STAG2 | 0.25 |
| BCLAF1 | 0.072 | CIC | 0.035 | FAT2 | 0.07 | NF1 | 0.017 | PIK3CA | 0.239 |
| SOX9 | 0.036 | PHF6 | 0.031 | NRAS | 0.033 | TBX3 | 0.01 | ATM | 0.096 |
| RB1 | 0.068 | NRAS | 0.047 | EPAS1 | 0.014 | SETD2 | 0.01 | ARID2 | 0.088 |
| IDH2 | 0.032 | PIK3CA | 0.035 | ATM | 0.056 | FAT1 | 0.03 | EGFR | 0.048 |
| CASP8 | 0.018 | BAP1 | 0.031 | LARP1 | 0.023 | MLL2 | 0.03 | CPS1 | 0.07 |
| CBFB | 0.014 | TNRC18 | 0.019 | NFE2L2 | 0.047 | MUC2 | 0.013 | FBXW7 | 0.066 |
| KIT | 0.027 | CDH1 | 0.012 | PKD1L1 | 0.023 | R3HDM1 | 0.013 | PTCH1 | 0.066 |
| BRAF | 0.018 | FAT2 | 0.012 | MAPK1 | 0.009 | FAM123B | 0.023 | ZNF750 | 0.059 |
| MLH1 | 0.05 | ATM | 0.012 | APC | 0.038 | TNRC18 | 0.023 | SMARCA4 | 0.103 |
| ERBB2 | 0.05 | BRCA1 | 0.012 | TET2 | 0.038 | KEAP1 | 0.01 | HLA-A | 0.048 |
| MAP2K4 | 0.045 | MAMLD1 | 0.012 | VHL | 0.033 | NUDT11 | 0.02 | MGA | 0.096 |
| TCF12 | 0.023 | LTBP3 | 0.012 | CPS1 | 0.033 | KDR | 0.017 | BRCA2 | 0.096 |
| KDR | 0.041 | TP53 | 0.012 | SHROOM4 | 0.033 | RET | 0.017 | PKD1L1 | 0.088 |
| CRIPAK | 0.032 | RET | 0.012 | HLA-B | 0.028 | MAPK1 | 0.007 | BRCA1 | 0.074 |
| P | | Q | | R | | S | | T | |
| TP53 | 0.723 | PBRM1 | 0.248 | TP53 | 0.244 | TP53 | 0.467 | PIK3CA | 0.433 |

*Continued on next page*

Supplementary Table 9 – *Continued from previous page*

| | | | | | | | | | |
|---|---|---|---|---|---|---|---|---|---|
| CDKN2A | 0.331 | VHL | 0.302 | STAG2 | 0.039 | CTNNB1 | 0.281 | CDH1 | 0.183 |
| MLL2 | 0.263 | BAP1 | 0.104 | ATRX | 0.051 | PIK3CA | 0.092 | MAP3K1 | 0.12 |
| CASP8 | 0.162 | SETD2 | 0.09 | SLC26A3 | 0.029 | BCLAF1 | 0.082 | FBXW7 | 0.026 |
| HRAS | 0.081 | MTOR | 0.075 | FAT1 | 0.037 | ARID2 | 0.138 | TBX3 | 0.047 |
| FAT2 | 0.098 | MET | 0.058 | PKD1L1 | 0.037 | MLL3 | 0.136 | ZFP36L1 | 0.022 |
| ATM | 0.064 | FAT2 | 0.019 | MUC2 | 0.034 | RNF43 | 0.044 | SMAD4 | 0.039 |
| MLL3 | 0.129 | NF2 | 0.037 | MTOR | 0.032 | EGFR | 0.061 | CBFB | 0.03 |
| PTCH1 | 0.059 | MUC2 | 0.029 | MLL2 | 0.029 | NSD1 | 0.104 | TNRC18 | 0.029 |
| FBXW7 | 0.112 | NOTCH1 | 0.027 | MAMLD1 | 0.029 | AR | 0.085 | ERBB2 | 0.013 |
| BRCA2 | 0.053 | ERCC6 | 0.027 | MAPK1 | 0.027 | ERBB2 | 0.041 | RUNX1 | 0.026 |
| NSD1 | 0.09 | KIAA2018 | 0.024 | PCF11 | 0.012 | CPS1 | 0.068 | NF1 | 0.021 |
| JUB | 0.087 | MLL3 | 0.022 | MGA | 0.022 | KIT | 0.031 | PAXIP1 | 0.019 |
| EPHA2 | 0.081 | NSD1 | 0.022 | NSD1 | 0.007 | BAP1 | 0.031 | TGFBR2 | 0.018 |
| WT1 | 0.039 | IL21R | 0.022 | BCLAF1 | 0.017 | NF1 | 0.061 | MGA | 0.018 |
| TNRC18 | 0.039 | HNF1B | 0.011 | CIC | 0.007 | KIAA2018 | 0.056 | SMAD2 | 0.008 |
| TGFBR2 | 0.076 | NF1 | 0.021 | KRAS | 0.007 | MTOR | 0.056 | TCF12 | 0.016 |
| FAM120C | 0.017 | CYP11B1 | 0.021 | GPR128 | 0.007 | PCF11 | 0.053 | RNF43 | 0.008 |
| NF1 | 0.067 | RB1 | 0.019 | PBRM1 | 0.015 | ZNF512B | 0.024 | GPS2 | 0.014 |
| HLA-A | 0.062 | DPCR1 | 0.019 | GPR50 | 0.015 | NOTCH1 | 0.048 | NUMBL | 0.005 |
| U | | V | | | | | | | |
| PIK3CA | 0.09 | BRAF | 0.173 | | | | | | |
| RB1 | 0.061 | RPTN | 0.019 | | | | | | |
| APC | 0.054 | ARID2 | 0.011 | | | | | | |
| TET2 | 0.015 | HRAS | 0.025 | | | | | | |
| MLL3 | 0.034 | SPOP | 0.024 | | | | | | |
| FAT1 | 0.029 | NRAS | 0.024 | | | | | | |
| EGFR | 0.028 | NF1 | 0.023 | | | | | | |
| NRAS | 0.011 | ATM | 0.023 | | | | | | |
| TNRC18 | 0.018 | FOXA1 | 0.011 | | | | | | |
| CYP11B1 | 0.009 | MLL2 | 0.021 | | | | | | |
| SMG7 | 0.008 | APC | 0.018 | | | | | | |
| PAXIP1 | 0.008 | NUDT11 | 0.015 | | | | | | |
| RHOA | 0.008 | LARP1 | 0.007 | | | | | | |
| ATM | 0.015 | MUC2 | 0.012 | | | | | | |
| PIK3R1 | 0.015 | CDK12 | 0.011 | | | | | | |
| ZNF512B | 0.007 | SHROOM4 | 0.011 | | | | | | |
| AR | 0.013 | PIK3CA | 0.01 | | | | | | |
| KIT | 0.013 | FBXW7 | 0.01 | | | | | | |
| SMAD4 | 0.012 | BCLAF1 | 0.009 | | | | | | |
| KDM6A | 0.011 | EMG1 | 0.009 | | | | | | |

Supplementary Table 10: Pathway enrichment (FDR $< 0.05$) of highly connected genes across all clusters (at least $22$ edges, or one per cluster on average) against KEGG pathways [39]. Pathways also significantly enriched in genes with $< 22$ edges across all clusters are not shown.

| Pathway | FDR |
|---|---|
| HIF-1 signaling pathway | $6.1 \times 10^{-08}$ |
| Th17 cell differentiation | $1.2 \times 10^{-05}$ |
| Apoptosis | $4.2 \times 10^{-04}$ |
| Melanogenesis | $6.2 \times 10^{-04}$ |
| Parathyroid hormone synthesis, secretion and action | $7.2 \times 10^{-04}$ |
| Long-term potentiation | $7.8 \times 10^{-04}$ |
| Jak-STAT signaling pathway | $8.8 \times 10^{-04}$ |
| p53 signaling pathway | $9.9 \times 10^{-04}$ |
| Homologous recombination | $1.3 \times 10^{-03}$ |
| Ubiquitin mediated proteolysis | $2.1 \times 10^{-03}$ |
| cAMP signaling pathway | $2.3 \times 10^{-03}$ |
| Fanconi anemia pathway | $2.9 \times 10^{-03}$ |
| Mismatch repair | $2.9 \times 10^{-03}$ |
| Lysine degradation | $3.7 \times 10^{-03}$ |
| Long-term depression | $3.8 \times 10^{-03}$ |
| Citrate cycle (TCA cycle) | $5.5 \times 10^{-03}$ |
| Adipocytokine signaling pathway | $5.7 \times 10^{-03}$ |
| AMPK signaling pathway | $5.7 \times 10^{-03}$ |
| Gap junction | $1.2 \times 10^{-02}$ |
| Oxytocin signaling pathway | $1.4 \times 10^{-02}$ |
| Hedgehog signaling pathway | $1.6 \times 10^{-02}$ |
| Progesteron-mediated oocyte maturation | $1.7 \times 10^{-02}$ |
| Toll-like receptor signaling pathway | $2.0 \times 10^{-02}$ |
| Tight junction | $2.1 \times 10^{-02}$ |
| Regulation of lipolysis in adipocytes | $2.1 \times 10^{-02}$ |
| TNF signaling pathway | $2.2 \times 10^{-02}$ |
| Glutathione metabolism | $2.3 \times 10^{-02}$ |
| Leukocyte transendothelial migration | $2.4 \times 10^{-02}$ |
| Serotonergic synapse | $2.5 \times 10^{-02}$ |
| Mitophagy | $3.2 \times 10^{-02}$ |
| RIG-I-like receptor signaling pathway | $3.9 \times 10^{-02}$ |

## Supplementary References


[1] Ciriello, G. *et al.* Emerging landscape of oncogenic signatures across human cancers. *Nature Genetics* **45**, 1127–1133 (2013).

[2] Hoadley, K. A. *et al.* Multiplatform analysis of 12 cancer types reveals molecular classification within and across tissues-of-origin. *Cell* **158**, 929–944 (2014).

[3] Nowell, P. C. The clonal evolution of tumor cell populations. *Science* **194**, 23–28 (1976).

[4] Hanahan, D. & Weinberg, R. A. Hallmarks of cancer: the next generation. *Cell* **144**, 646–674 (2011).

[5] Vogelstein, B. *et al.* Genetic alterations during colorectal tumor development. *New England Journal of Medicine* **319**, 525–532 (1988).

[6] Desper, R. *et al.* Inferring tree models for oncogenesis from comparative genome hybridization data. *Journal of Computational Biology* **6**, 37–51 (1999).

[7] Beerenwinkel, N. *et al.* Learning multiple evolutionary pathways from cross-sectional data. *Journal of Computational Biology* **12**, 584–598 (2005).

[8] Beerenwinkel, N., Eriksson, N. & Sturmfels, B. Conjunctive Bayesian networks. *Bernoulli* **13**, 893–909 (2007).

[9] Gerstung, M., Baudis, M., Moch, H. & Beerenwinkel, N. Quantifying cancer progression with conjunctive Bayesian networks. *Bioinformatics* **25**, 2809–2815 (2009).

[10] Hainke, K., Rahnenführer, J. & Fried, R. Cumulative disease progression models for cross-sectional data: A review and comparison. *Biometrical Journal* **54**, 617–640 (2012).

[11] Attolini, C. S.-O. *et al.* A mathematical framework to determine the temporal sequence of somatic genetic events in cancer. *Proceedings of the National Academy of Sciences* **107**, 17604–17609 (2010).

[12] Misra, N., Szczurek, E. & Vingron, M. Inferring the paths of somatic evolution in cancer. *Bioinformatics* **30**, 2456–2463 (2014).

[13] Farahani, H. S. & Lagergren, J. Learning oncogenetic networks by reducing to mixed integer linear programming. *PLoS ONE* **8**, e65773 (2013).

[14] Ramazzotti, D. *et al.* CAPRI: efficient inference of cancer progression models from cross-sectional data. *Bioinformatics* **31**, 3016–3026 (2015).

[15] Leiserson, M. D., Wu, H.-T., Vandin, F. & Raphael, B. J. Comet: a statistical approach to identify combinations of mutually exclusive alterations in cancer. *Genome Biology* **16**, 160 (2015).

[16] Constantinescu, S., Szczurek, E., Mohammadi, P., Rahnenführer, J. & Beerenwinkel, N. TiMEx: a waiting time model for mutually exclusive cancer alterations. *Bioinformatics* **32**, 968–975 (2015).

[17] Babur, Ö. *et al.* Systematic identification of cancer driving signaling pathways based on mutual exclusivity of genomic alterations. *Genome Biology* **16**, 45 (2015).

[18] Kim, Y.-A., Cho, D.-Y., Dao, P. & Przytycka, T. M. MEMCover: integrated analysis of mutual exclusivity and functional network reveals dysregulated pathways across multiple cancer types. *Bioinformatics* **31**, i284–i292 (2015).

[19] Jerby-Arnon, L. *et al.* Predicting cancer-specific vulnerability via data-driven detection of synthetic lethality. *Cell* **158**, 1199–1209 (2014).

[20] Cristea, S., Kuipers, J. & Beerenwinkel, N. pathTiMEx: Joint inference of mutually exclusive cancer pathways and their progression dynamics. *Journal of Computational Biology* **24**, 603–615 (2017).

[21] TCGA Research Network. Comprehensive molecular characterization of urothelial bladder carcinoma. *Nature* **507**, 315–322 (2014).

[22] TCGA Network. Comprehensive molecular portraits of human breast tumours. *Nature* **490**, 61–70 (2012).

[23] Ciriello, G. *et al.* Comprehensive molecular portraits of invasive lobular breast cancer. *Cell* **163**, 506–519 (2015).

[24] TCGA Research Network *et al.* The cancer genome atlas pan-cancer analysis project. *Nature Genetics* **45**, 1113–1120 (2013).

[25] TCGA Network. Comprehensive molecular characterization of human colon and rectal cancer. *Nature* **487**, 330–337 (2012).

[26] Brennan, C. W. *et al.* The somatic genomic landscape of glioblastoma. *Cell* **155**, 462–477 (2013).

[27] TCGA Network. Comprehensive genomic characterization of head and neck squamous cell carcinomas. *Nature* **517**, 576–582 (2015).

[28] TCGA Research Network. Comprehensive molecular characterization of clear cell renal cell carcinoma. *Nature* **499**, 43–49 (2013).

[29] TCGA Research Network. Genomic and epigenomic landscapes of adult de novo acute myeloid leukemia. *New England Journal of Medicine* **368**, 2059–2074 (2013).

[30] TCGA Research Network. Comprehensive, integrative genomic analysis of diffuse lower-grade gliomas. *New England Journal of Medicine* **372**, 2481–2498 (2015).

[31] TCGA Research Network. Comprehensive molecular profiling of lung adenocarcinoma. *Nature* **511**, 543–550 (2014).

[32] TCGA Research Network. Comprehensive genomic characterization of squamous cell lung cancers. *Nature* **489**, 519–525 (2012).

[33] TCGA Research Network. Integrated genomic analyses of ovarian carcinoma. *Nature* **474**, 609–615 (2011).

[34] TCGA Research Network. Integrated genomic characterization of endometrial carcinoma. *Nature* **497**, 67–73 (2013).

[35] Spirtes, P., Glymour, C. N. & Scheines, R. *Causation, prediction, and search* (MIT Press, 2000).

[36] Kalisch, M., Mächler, M., Colombo, D., Maathuis, M. H. & Bühlmann, P. Causal inference using graphical models with the R package pcalg. *Journal of Statistical Software* **47**, 1–26 (2012).

[37] Chickering, D. M. Optimal structure identification with greedy search. *Journal of Machine Learning Research* **3**, 507–554 (2002).

[38] Suter, P. & Kuipers, J. BiDAG: Software for the efficient inference and sampling of Bayesian networks (2017). https://CRAN.R-project.org/package=BiDAG.

[39] Kanehisa, M., Furumichi, M., Tanabe, M., Sato, Y. & Morishima, K. KEGG: new perspectives on genomes, pathways, diseases and drugs. *Nucleic Acids Research* **45**, D353–D361 (2016).
